# Untargeted metabolomics reveals the effects of pre-analytic storage on serum metabolite profiles from healthy cats

**DOI:** 10.1371/journal.pone.0303500

**Published:** 2024-05-30

**Authors:** Patrick C. Barko, Anisha Jambhekar, Kelly S. Swanson, Marcella D. Ridgway, David A. Williams

**Affiliations:** 1 Department of Veterinary Clinical Medicine, College of Veterinary Medicine, University of Illinois at Urbana-Champaign; Urbana, Illinois, United States of America; 2 Fuqua School of Business, Duke University, Durham, North Carolina, United States of America; 3 Department of Animal Sciences and Division of Nutritional Sciences, College of Agricultural, Consumer and Environmental Sciences, University of Illinois at Urbana-Champaign; Urbana, Illinois, United States; University of California Riverside, UNITED STATES

## Abstract

Untargeted metabolomics investigations have characterized metabolic disturbances associated with various diseases in domestic cats. However, the pre-analytic stability of serum metabolites in the species is unknown. Our objective was to compare serum metabolomes from healthy cats stored at -20°C for up to 12 months to samples stored at -80°C. Serum samples from 8 adult, healthy cats were stored at -20°C for 6 months, -20°C for 12 months, or -80°C for 12 months. Untargeted liquid chromatography-mass spectrometry was used to generate serum metabolite profiles containing relative abundances of 733 serum metabolites that were compared among storage conditions. Unsupervised analysis with principal component analysis and hierarchical clustering of Euclidian distances revealed separation of samples from individual cats regardless of storage condition. Linear mixed-effects models identified 75 metabolites that differed significantly among storage conditions. Intraclass correlation analysis (ICC) classified most serum metabolites as having excellent (ICC ≥ 0.9; 33%) or moderate (ICC 0.75–0.89; 33%) stability, whereas 13% had poor stability (ICC < 0.5). Biochemicals that varied significantly among storage conditions and classified with poor stability included glutathione metabolites, amino acids, gamma-glutamyl amino acids, and polyunsaturated fatty acids. The benzoate; glycine, serine and threonine; tryptophan; chemical (xenobiotics); acetylated peptide, and primary bile acid sub pathways were enriched among highly stable metabolites, whereas the monohydroxy fatty acid, polyunsaturated fatty, and monoacylglycerol sub-pathways were enriched among unstable metabolites. Our findings suggest that serum metabolome profiles are representative of the cat of origin, regardless of storage condition. However, changes in specific serum metabolites, especially glutathione, gamma-glutamyl amino acid, and fatty acid metabolites were consistent with increased sample oxidation during storage at -20°C compared with -80°C. By investigating the pre-analytic stability of serum metabolites, this investigation provides valuable insights that could aid other investigators in planning and interpreting studies of serum metabolomes in cats.

## Introduction

Untargeted metabolomics investigations aim to detect and quantify all small molecules (<1500 kDa) present in an organism, tissue, cell, or biologic sample in an unbiased manner [1, 2]. These biochemicals are precursors, intermediates, and end-products of endogenous and exogenous (microbial, dietary, other environmental) metabolic processes. Analysis of metabolomic profiles can be used to characterize metabolic phenotypes (metabotypes) associated with diseases or other experimental conditions. Untargeted metabolomics experiments use high-throughput methods, typically nuclear magnetic resonance imaging (NMR) or liquid chromatography/mass spectrometry (LMCS), to measure biochemicals in an unbiased manner. Elucidation of metabolic disturbances can aid in generating novel hypotheses and discovery of biomarkers associated with disease states or experimental perturbations.

According to a recent demographic survey conducted by the American Veterinary Medical Association, there are greater than 60 million pet cats in the United States alone, and veterinary medical care and nutrition for domestic cats support multi-billion-dollar industries [3]. Additionally, domestic cats are valuable model species for the investigation of diseases in humans [4–7]. Untargeted metabolomics investigations have been utilized to study the influence of nutrition and various diseases including diabetes mellitus, obesity, chronic renal disease, mammary carcinoma, and exocrine pancreatic insufficiency on circulating metabolomes in cats [8–14]. One advantage of using cats as a model species for human diseases is that cohabitation with humans ensures they are exposed to similar environmental factors. Owing to this, and cats’ popularity as companion animals, most investigations of feline diseases require that samples are collected by veterinarians in primary care settings. Despite the growing popularity of untargeted metabolomics studies, the pre-analytic stability of metabolites in serum samples exposed to storage in clinical freezers has not been studied in cats. Understanding the stability of metabolites in serum is especially important for studies utilizing samples collected in veterinary clinics or field settings where samples may be stored in sub-optimal thermal conditions due to the unavailability of laboratory freezers. Understanding how interim storage in clinical freezers (-20°C) compared with laboratory storage (-80°C) affects serum metabolite profiles will aid in the interpretation of untargeted metabolomics studies involving samples collected by veterinary researchers and stored in a clinical setting where ultra-low temperature freezers are unavailable.

The purpose of this study was to assess the stability of serum metabolite profiles in samples from cats exposed to interim storage at -20°C for 6 and 12 months, compared to samples stored at -80°C. We used an untargeted metabolomics approach to quantify the relative abundances of serum metabolites via high-performance liquid chromatography and tandem mass spectrometry (UPLC-MS/MS). We hypothesized that extended storage at -20°C, compared with -80°C would alter serum metabolite profiles in serum samples from healthy cats. Consistent with our hypothesis, we identified changes in the relative abundance of serum metabolites associated with storage at -20°C. These findings could aid investigators in understanding the impact of pre-analytic storage on untargeted serum metabolite profiles in cats.

## Materials and methods

### Ethics statement

All sample collection methods were approved by the Institutional Animal Care and Use Committee (protocol ID 14256).

### Sample collection and storage

Serum samples from 8 healthy, adult, male castrated cats from a research colony were used in this study. All cats were approximately 9 years-old at the time of sample collection. Blood samples were collected by jugular venipuncture after an overnight fast. The blood samples were collected into empty, sterile tubes and allowed to clot at room temperature prior to centrifugation at 2500 rpm for 10 minutes. The resulting serum was decanted and aliquoted into three empty, sterile, polypropylene tubes. One aliquot from each cat was transferred to an ultra-low temperature freezer and stored at -80°C. The remaining two aliquots were placed in a standard clinical freezer and stored at -20°C. The two aliquots stored at -20°C were transferred to -80°C after 6 and 12 months, respectively. After 12 months, all samples were submitted for untargeted metabolomic analysis.

### Untargeted quantification of serum metabolites

Untargeted serum metabolite profiles were generated via ultra-high performance liquid chromatography-tandem mass spectroscopy (UPLC-MS/MS) by a commercial laboratory (Metabolon Inc.; Morrisville, NC) as previously described [15, 16]. Serum samples (100 μL) were deproteinated using methanol (500 μL) precipitation with vigorous shaking for 2 min followed by centrifugation. The methanol contained four recovery standards (DL-2-fluorophenylglycine, tridecanoic acid, d6-cholesterol, and 4-chlorophenylalanine) used to assess extraction efficiency. The resulting extracts were dried and reconstituted in solvents compatible to each of the four UPLC-MS/MS methods: 50 μL of 6.5 mM ammonium bicarbonate in water (pH 8) for negative ion analysis methods and 50 μL 0.1% formic acid in water (pH 3.5) for the positive ion analysis methods. The resulting extracts were separated into fractions and subjected to four methods for quantification of chemically diverse compounds: two for reverse phase (RP)/UPLC-MS/MS with positive ion mode electrospray ionization (ESI), one for analysis by RP/UPLC-MS/MS with negative ion mode ESI, and one for analysis by HILIC/UPLC-MS/MS with negative ion mode ESI. All methods utilized a Waters ACQUITY ultra-performance liquid chromatographer and a Thermo Scientific Q-Exactive high resolution/accurate mass spectrometer interfaced with a heated electrospray ionization source and Orbitrap mass analyzer operated at 35,000 mass resolution. The MS analysis alternated between MS and data-dependent MSn scans using dynamic exclusion. The scan range varied slighted between methods but covered 70–1000 m/z. Analytic controls consisting of pooled samples, generated from small volumes of each experimental sample, were analyzed simultaneously with experimental samples. Methanol-extracted water samples served as process blanks and quality-control standards that were known to not interfere with the measurement of endogenous compounds were spiked into every analyzed sample, allowing monitoring of instrument performance, and aiding chromatographic alignment. Experimental samples were randomized across the platform run with quality control samples spaced evenly among the injections. The injection volumes were 5 μL. Additional methodological details, including the gradient profiles and quality control standards, are presented in the S1 File. Data extraction, relative metabolite quantification, and compound identification were performed using a proprietary software platform by Metabolon Inc. Compounds were identified by comparison to library entries of purified and authenticated standards. Metabolites were quantified by measuring the area-under-the-curve of the chromatographic peak. Metabolite abundance data were normalized by median scaling and missing values were imputed with the sample set minimum.

### Statistical analysis

All statistics were performed in the R language for statistical computing (version 4.03). The serum metabolite dataset, including the raw and normalized/imputed data, and R code used in these analyses are available in our GitHub repository (https://github.com/pcbarko/Barko-Feline-Serum-Metabolome-Stability). The median scaled data were log-transformed prior to statistical analysis. For unsupervised analysis, principal component analysis (PCA) and hierarchical clustering of Euclidian distances were performed. Linear mixed-effects models were used to detect significant differences in serum metabolites between the three storage conditions. The dependent variables were measurements of relative abundances of individual metabolites, the fixed effects were the storage conditions, and the identity of the cat of origin for each sample was the random effect. Post-hoc, pairwise comparisons of metabolites that varied significantly in the mixed-effects models utilized least square means tests.

For each serum metabolite, intraclass correlation coefficients (ICC) were calculated to evaluate variability in measurements from replicate samples stored under different pre-analytic conditions. ICC is a measure of variability defined by the ratio of between-subject variance to the total variance (sum of between-subject and within-subject variance) that has been used to assess the repeatability of serum metabolite measurements in metabolomics studies with repeated measures [3–6]. ICC was calculated from the linear mixed-effects models using the “performance” R package [7]. The stability of serum metabolite abundances was classified based on the ICC as follows: excellent (ICC ≥ 0.90), moderate (ICC 0.75–0.89), fair (ICC 0.5–0.74), and poor (ICC < 0.50).

Metabolite set enrichment analysis (MSEA) was used to identify metabolic sub-pathways enriched in metabolites with excellent or poor stability. Metabolites were ranked by the ICC in decreasing order, so those classified with excellent stability (ICC approaching 1) were at the top and those classified with poor stability (ICC approaching 0) were at the bottom of ranked vector of metabolites. Next, MSEA was implemented by using the “fgsea” R package to calculate normalized enrichment scores (NES) for each metabolic sub-pathway [8]. A positive NES indicates that a sub-pathway is enriched in highly stable metabolites, whereas a negative NES indicates that a sub-pathway is enriched in metabolites with lower stability.

For tests utilizing multiple comparisons, p-values were adjusted using the Benjamini-Hochberg method to control false discovery (q-values) [9, 10]. Results with q-values < 0.05 were considered statistically significant.

## Results and discussion

The purpose of this investigation was to assess the stability of serum metabolomes from healthy cats exposed to pre-analytic storage at -20°C for 6 and 12 months, compared to ultra-low temperature storage at -80°C. Despite the increasing number of studies utilizing untargeted metabolomics, there have been few investigations of the pre-analytic stability of serum metabolites during extended storage, and none have been conducted in cats.

**Fig 1:**
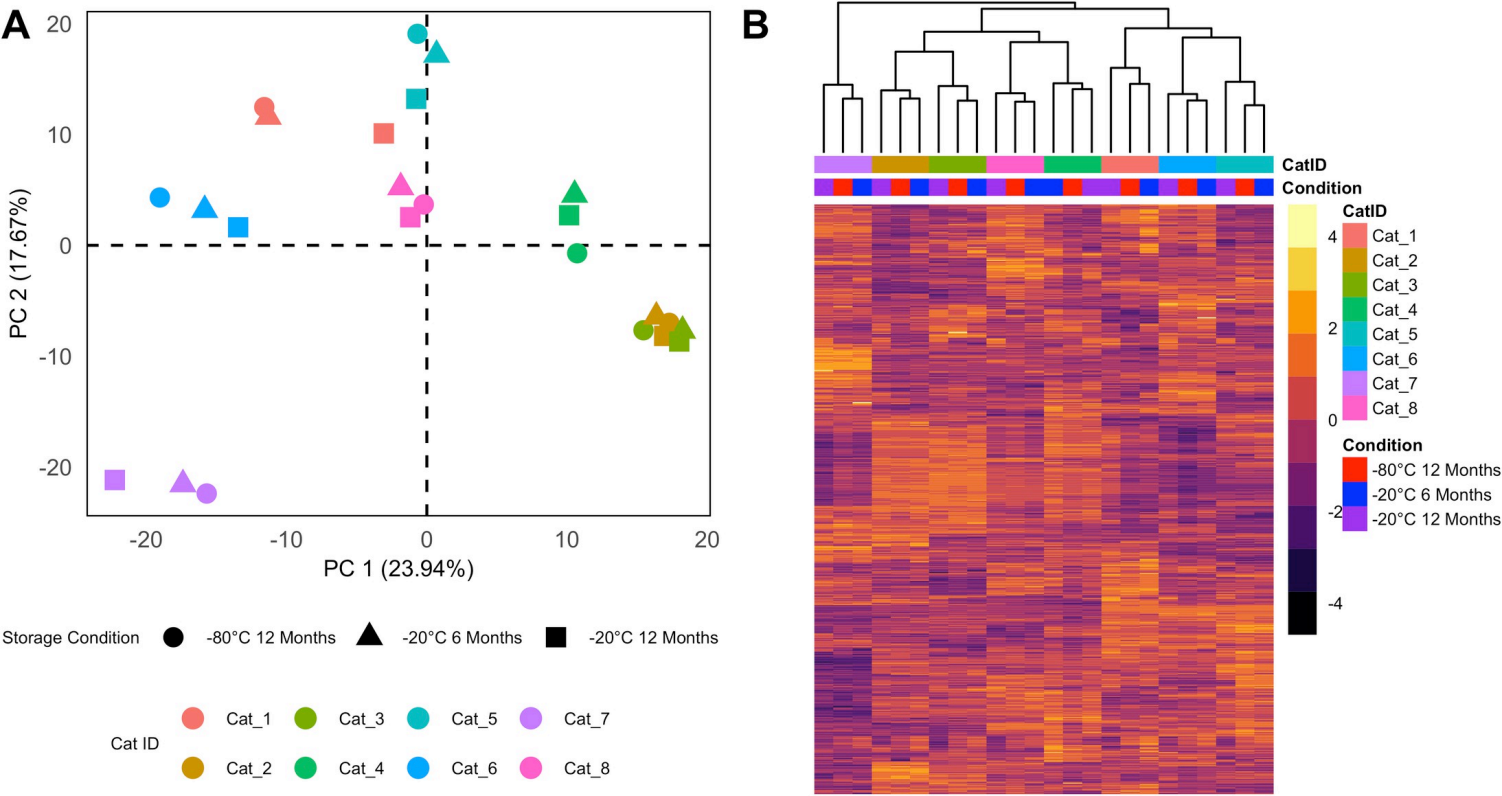
Unsupervised analysis of serum metabolomes. **A)** Principal component analysis. In the PCA score plot, points represent each sample. Each sample is colored according to the individual cat of origin, such that samples from the same cat are represented by the same color. The shape of each point represents the storage condition to which the samples were exposed. **B)** Statistical heatmap of serum metabolites. A statistical heatmap was used to visualize the relative abundance of serum metabolites. The color of each cell is proportional to the z-scaled relative abundance of each metabolite. Individual metabolites (rows) are organized by hierarchical clustering of Euclidian distances, but the dendrogram is not shown. Samples (columns) are organized by hierarchical clustering of Euclidian distances and the clustering dendrogram is present above along the x-axis at the top of the image.

We measured 733 named metabolites in the sera of 8 healthy cats. The LC/MS/MS negative method yielded detection of 272 metabolites, the positive early method yielded detection of 209 metabolites, the LC/MS/MS positive late method yielded detection of 184 metabolites, and the LC/MS/MS polar method yielded detection of 68 metabolites (S2 File in S1 File). Metabolites were organized into 8 metabolic super-pathways detected and lipid metabolites were the most numerous (n = 348), followed by amino acids (n = 178), xenobiotics (n = 71), nucleotides (n = 48), peptides (n = 31), cofactors and vitamins (n = 26), carbohydrates (n = 22), and energy (n = 9). Unsupervised analysis using PCA revealed separation of sample aliquots according to the individual cat of origin (Fig 1A). No clear pattern of separation was observed in relation to storage condition. However, within the clusters formed by samples from individual cats, there was apparent separation of some samples stored at -20°C for 12 months from those stored at -20°C for 6 months and -80°C. Though this was only observed in 3/8 cats (cats 1, 5, and 7), it is consistent with changes in the serum metabolomes during extended storage at -20°C. It may also suggest that some individual cats’ serum metabolome profiles are more unstable than others when exposed to storage at -20°C. Consistent with the results of PCA, a heatmap of relative metabolite abundances with hierarchical clustering of samples based on their Euclidian distances showed that samples from the same cats clustered together regardless of storage condition (Fig 1B). The PCA and hierarchical clustering results indicate that inter-individual variation among cats is greater than intra-individual variation among different storage conditions. Our results are consistent with a previous untargeted metabolomics study of human serum wherein intra-individual variation was smaller than inter-individual variation, regardless of pre-analytic processing and storage conditions [11].

**Fig 2:**
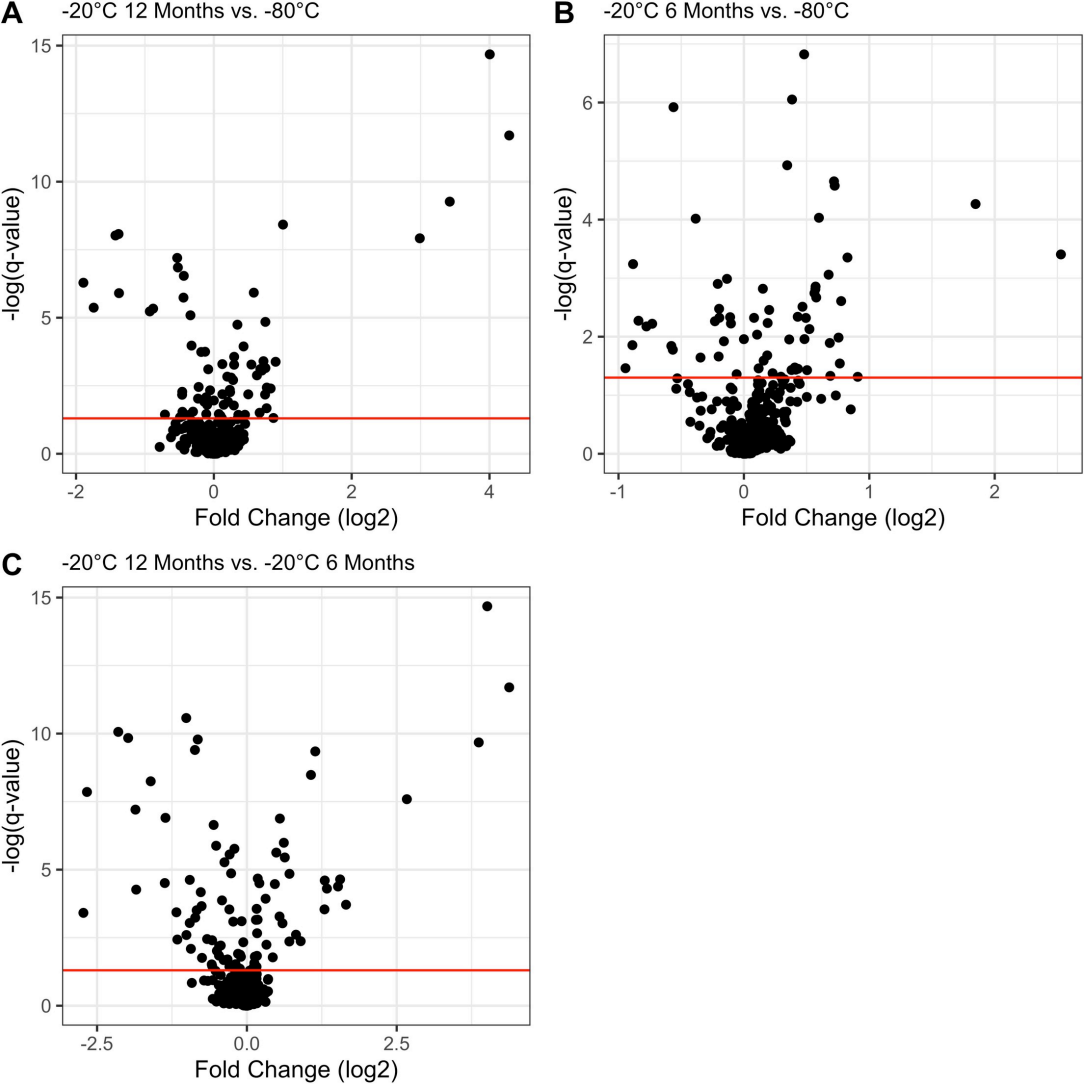
Volcano plots of statistical contrasts. Volcano plots of the -log of the q-values (y-axis) and the log2 of the fold-change between conditions were generated for each statistical contrast in the post-hoc tests among storage conditions. **A)** Volcano plot for the comparison of samples stored at -20°C for 12 months and -80°C. **B)** Volcano plot for the comparison of samples stored at -20°C 6 Months and -80°C. C) Volcano plot for the comparison of samples stored at -20°C for 12 months and -20°C for 6 months. The red horizontal lines represent the threshold for statistical significance (q-value < 0.05) and all metabolites above this line were significantly variable.

Linear mixed effects models identified 75 serum metabolites with relative abundances that varied significantly (q-value <0.05) among storage conditions. Sixty-one metabolites varied significantly between samples stored at 20°C for 12 months and -80°C (Fig 2A). Fifty-five metabolites varied significantly between samples stored at 20°C for 6 months and -80°C (Fig 2B). Sixty-eight metabolites varied significantly between samples stored at 20°C for 12 months and -20°C for 6 months (Fig 2C).

**Fig 3:**
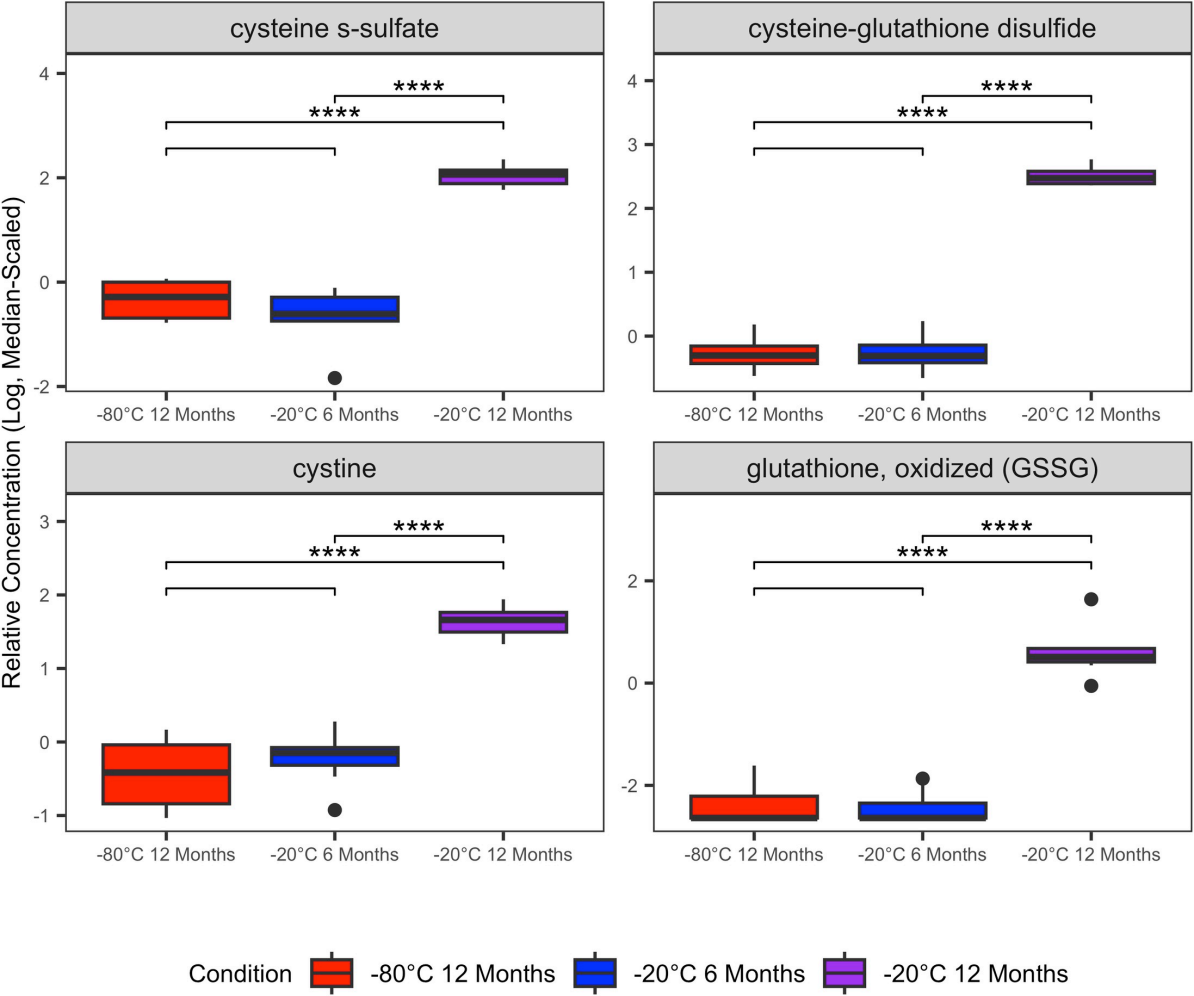
Significantly variable serum glutathione metabolites. Relative abundances of serum glutathione metabolites were increased in serum samples stored at -20°C. Boxplots contains data from 8 individual cats with three biologic replicates from each cat representing the three different storage conditions. Linear mixed-effects models were used to identify serum metabolites that varied significantly among the storage conditions. Plots are annotated with the false discovery rate-adjusted p-values (q-values) derived from the post-hoc pairwise comparisons among storage conditions: q < 0.05 (*); q ≤ 0.01 (**); q ≤ 0.001 (***); q≤ 0.0001 (****). Non-significant comparisons are hidden.

**Fig 4:**
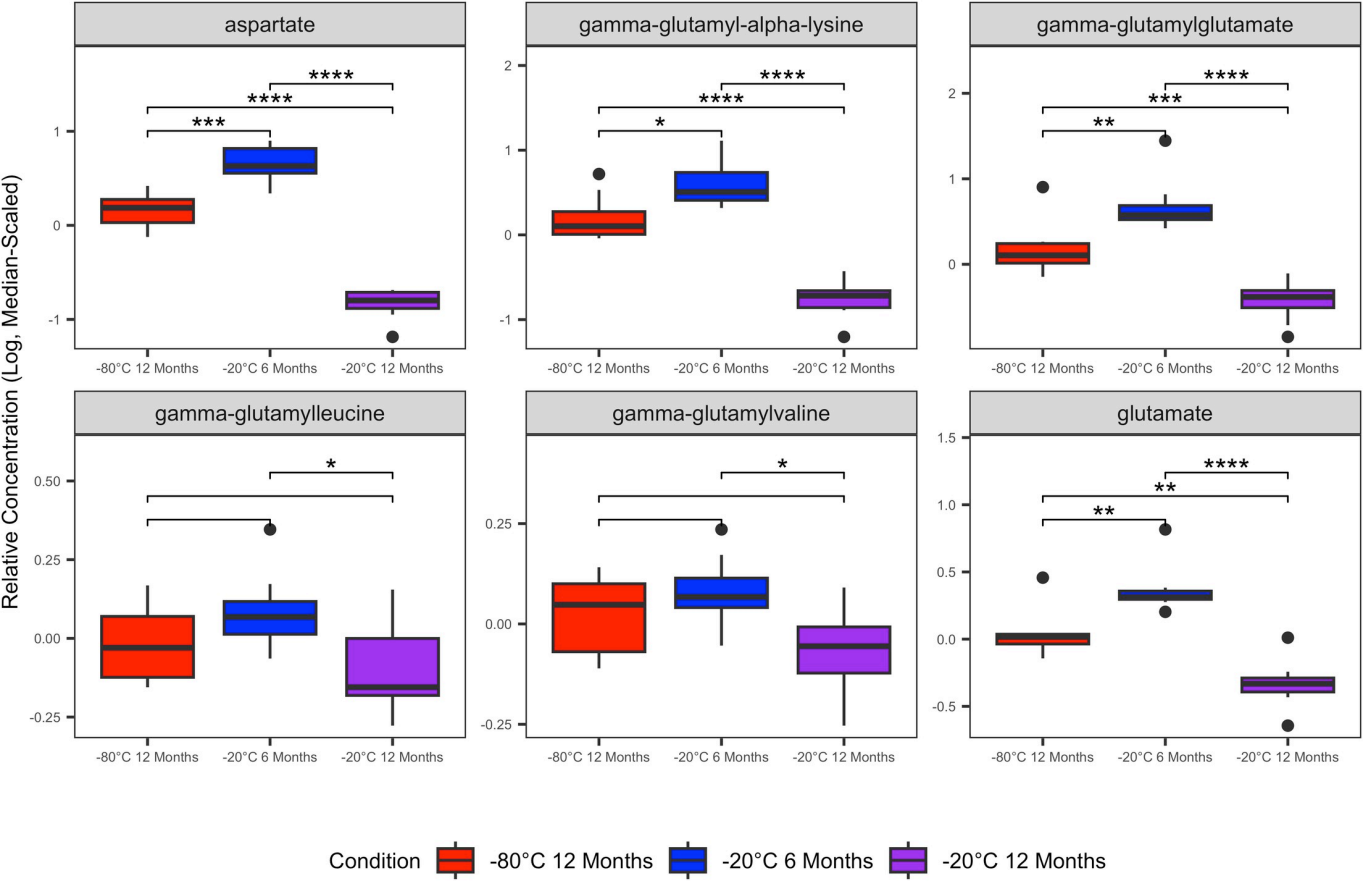
Significantly variable serum amino acid and gamma-glutamyl amino acid metabolites. Relative abundances of serum amino acid and gamma-glutamyl amino acid metabolites were decreased in serum samples stored at -20°C. Boxplots contains data from 8 individual cats with three biologic replicates from each cat representing the three different storage conditions. Linear mixed-effects models were used to identify serum metabolites that varied significantly among the storage conditions. Plots are annotated with the false discovery rate-adjusted p-values (P_adj_) derived from the post-hoc pairwise comparisons among storage conditions: q < 0.05 (*); q ≤ 0.01 (**); q ≤ 0.001 (***); q≤ 0.0001 (****). Non-significant comparisons are hidden.

**Fig 5:**
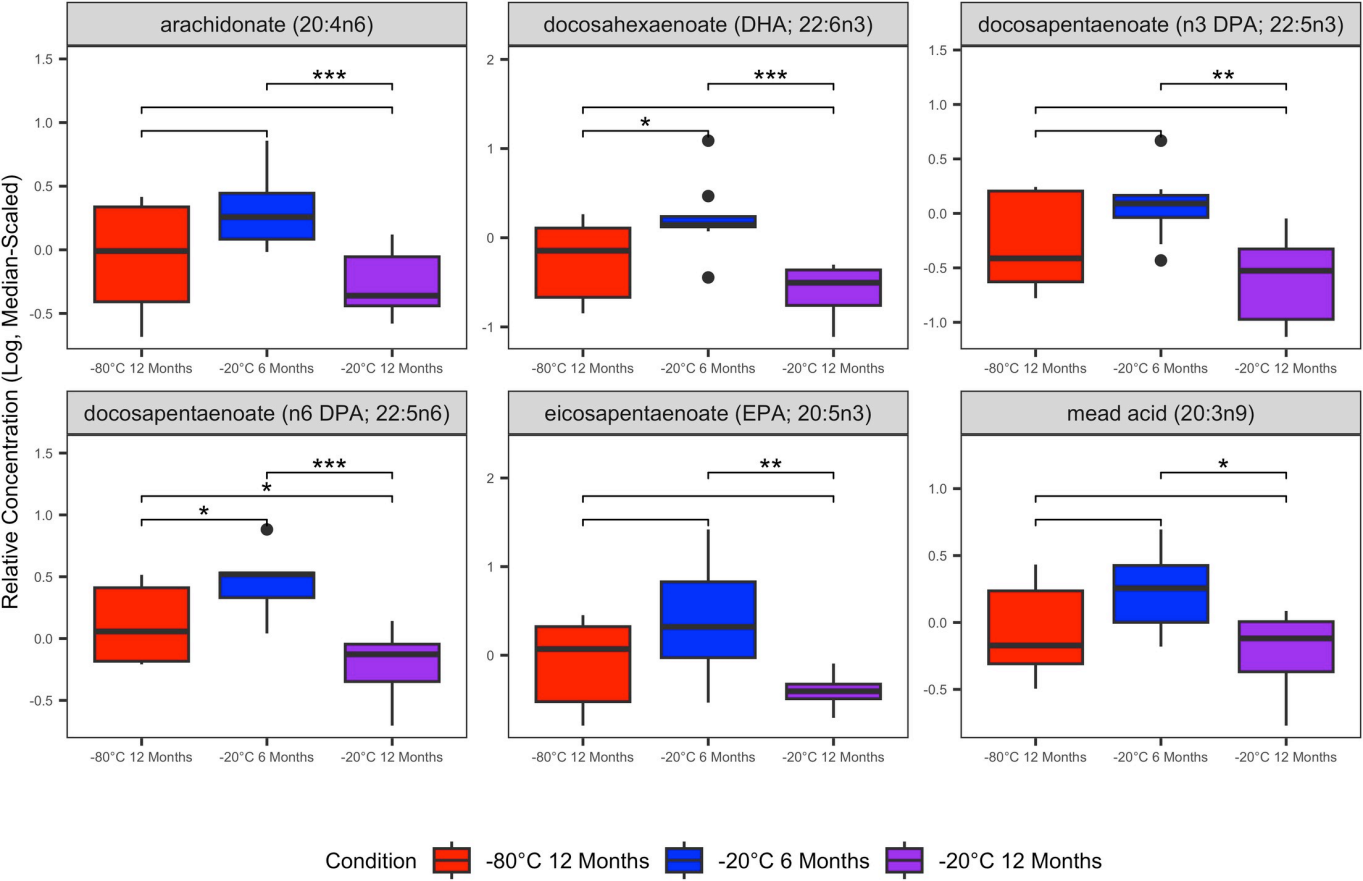
Significantly variable serum polyunsaturated fatty acid metabolites. Relative abundances of serum PUFA metabolites were decreased in serum samples stored at -20°C. Boxplots contains data from 8 individual cats with three biologic replicates from each cat representing the three different storage conditions. Linear mixed-effects models were used to identify serum metabolites that varied significantly among the storage conditions. Plots are annotated with the false discovery rate-adjusted p-values (q-values) derived from the post-hoc pairwise comparisons among storage conditions: q < 0.05 (*); q ≤ 0.01 (**); q ≤ 0.001 (***); q≤ 0.0001 (****). Non-significant comparisons are hidden.

Featured among significantly variable metabolites were several glutathione, amino acid, gamma-glutamyl amino acid, and polyunsaturated fatty acid metabolites. Glutathione metabolites cystine, cysteine-glutathione disulfide, cysteine-s-sulfate, and oxidized glutathione (GSSG) were increased in samples stored at -20°C for 12 months compared with samples stored at -80°C or -20°C for 6 months (Fig 3). Aspartate, glutamate, and several gamma-glutamyl amino acids were decreased significantly in samples stored at -20°C for 12 months compared with other storage conditions (Fig 4). Polyunsaturated fatty acids (PUFA) were decreased significantly in samples stored at -20°C for 12 months compared with other storage conditions (Fig 5). Complete output from the linear mixed effects models (S3 File S1 File), all post-hoc group-wise comparisons among storage conditions for significantly variable metabolites (S4 File S1 File), and boxplots of all serum metabolites that varied significantly in the linear mixed effects model (S5 File S1 File) are in the Supporting Information.

**Fig 6:**
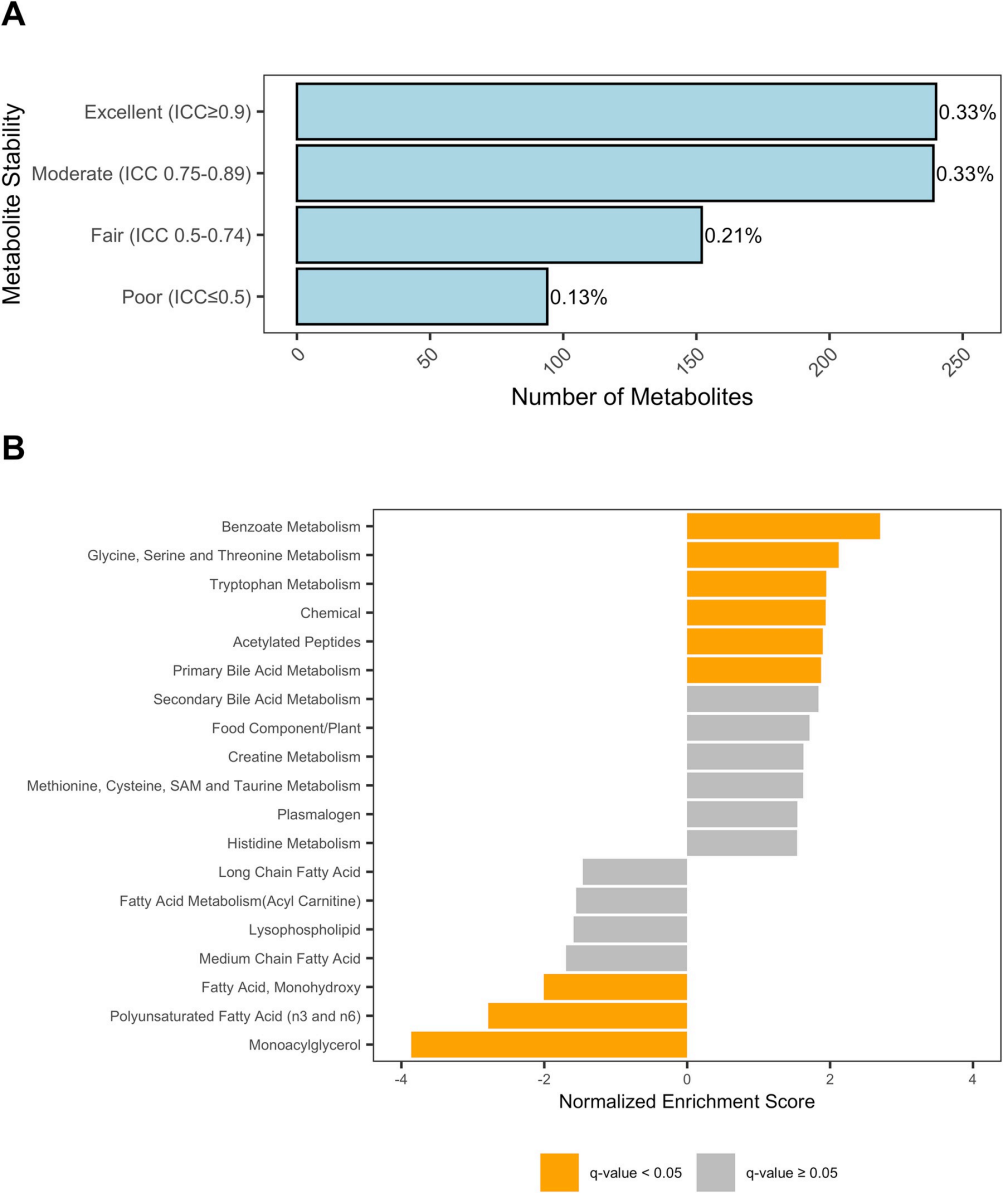
Analysis of metabolite stability. **A)** Intraclass correlation (ICC) statistics. ICC analysis was used to classify metabolites according to the degree of correlation among measurement of samples from the same cats stored under different conditions. The counts and proportions of metabolites classified with “excellent,” “moderate,” “fair,” or “poor” stability are represented. **B)** Metabolite set enrichment analysis. Metabolite set enrichment analysis (MSEA) was used to detect metabolic sub-pathways enriched among metabolites with high (positive NES) and low (negative NES) stability. Sub-pathways presented here are those with NES P > 0.05. Bars are annotated by the false discovery rate-adjusted p-value (q-value) such that grey bars represent pathways with q ≥ 0.05 and orange bars representing sub-pathways with q < 0.05.

Intraclass correlation coefficients (ICC) derived from the linear mixed-effects models were used to quantify variability in the relative abundances of serum metabolite in samples collected from the same individual cats but exposed to different pre-analytic storage conditions (full results in S6 File S1 File). Previous studies have used ICC to estimate the repeatability of serum metabolites measurements under different pre-analytic conditions [12–16]. The ICC represents the magnitude of correlation among relative metabolite abundances in samples from the same cat and was used to here as a proxy for metabolite stability. Highly stable metabolites will have a high degree of correlation among storage conditions in samples from the same cat, whereas poorly stable metabolites will have a low degree of correlation. Like correlation coefficients, ICC ranges from 0–1, with intra-class agreement among samples increasing as ICC approaches 1. The median ICC was 0.84 with an interquartile range of 0.68–0.92. Serum metabolite stability was classified as excellent (ICC≥0.9) in 33%, moderate (ICC 0.75–0.89) in 33%, fair (ICC 0.5–0.74) in 21%, and poor (ICC<0.5) in 13% of metabolites (Fig 6A). The ten most stable metabolites were 4-vinylphenol sulfate, 4-vinylguaiacol sulfate, phenol sulfate, 2-piperidinone, trimethylamine N-oxide, indolepropionate, 3-phenylpropionate (hydrocinnamate), p-cresol sulfate, serine, and stachydrine. The ten least stable metabolites were pterin, glycochenodeoxycholate, 2-docosahexaenoylglycerol (22:6), pseudouridine, palmitoyl ethanolamide, N-carbamoylsarcosine, glycochenodeoxycholate sulfate, glucuronate, beta-alanine, 2-stearoyl-GPE (18:0).

Using MSEA we identified significant enrichment of the benzoate (NES = 2.70; P_adj_ <0.001); glycine, serine and threonine (NES = 2.12; P_adj_ = 0.009); tryptophan (NES = 1.94; P_adj_ = 0.039); chemical (NES = 1.94; P_adj_ value = 0.031); acetylated peptides (NES = 1.90; P_adj_ = 0.045), and primary bile acid (NES = 1.87; P_adj_ = 0.049) sub-pathways among metabolites with higher stability (Fig 6B). Conversely, we identified enrichment of the monohydroxy fatty acid (NES = -2.0; P_adj_<0.043), polyunsaturated fatty acid (NES = -2.78; P_adj_<0.001), and monoacylglycerol (NES = -3.86; P_adj_<0.001) sub-pathways among metabolites with lower stability (Fig 6B). The complete output of MSEA is in the S7 File S1 File.

Many of the serum biochemicals that we identified as varying significantly among storage conditions that were also classified with poor stability in ICC analysis are involved in cellular redox reactions (e.g., glutathione metabolites, gamma-glutamyl amino acids). Consistent with increased sample oxidation during storage at -20°C, cystine, cysteine-glutathione disulfide, cysteine-s-sulfate, and oxidized glutathione (GSSG) were increased with extended storage of serum at -20°C (Fig 3). Glutathione (GSH) is generated either by recycling of GSSG or though the gamma-glutamyl amino acid cycle [17, 18]. In the gamma-glutamyl amino acid cycle extracellular amino acids are transported across the cellular membrane and converted to gamma-glutamyl amino acids by gamma-glutamyl aminotransferase (GGT). Subsequently, gamma-glutamyl amino acids are converted to glutamate, gamma-glutamyl cysteine, and finally to GSH which is either consumed in intracellular redox reactions or exported extracellularly. As GSH is oxidized to GSSG, it is plausible that precursors and intermediates in the regeneration of GSH are depleted in the sera. This interpretation is supported by decreased gamma-glutamyl amino acids, 5-oxoproline, and glutamate in the serum samples stored at -20°C (Fig 4). Additionally, many fatty acid oxidative substrates (e.g. PUFAs) were decreased in samples stored at -20°C and enriched in metabolic pathways with low stability (Figs 5 and 6B). Enrichment of fatty acid sub-pathways among serum metabolites with low stability is also consistent with increased oxidation with extended storage at -20°C as fatty acids are substrates for oxidation reactions [19].

We observed divergent patterns of stability among metabolites within the same metabolic sub-pathways. Examples of this include metabolites in the “alanine and aspartate metabolism,” “benzoate metabolism,” “diacylglycerol,” “endocannabinoid,” “BCAA metabolism,” “acyl carnitines,” “tyrosine metabolism,” “TCA cycle,” “sphingolipid metabolism,” “polyunsaturated fatty acid (n3 and n6),” “methionine, cysteine, SAM and taurine metabolism,” “lysophospholipids,” and “glutamate metabolism.” These sub-pathways contain metabolites that range from excellent to poor stability. Though metabolites in the same sub-pathway are presumed to be involved in similar biologic processes, they likely differ in their chemical properties and reactivity. It is also likely that enzymes present in serum that catalyze reactions involving specific subsets of metabolites are inactivated under different thermal conditions. As this was an untargeted, hypothesis-generating investigation we cannot determine the mechanisms that explain differential stability of metabolites within the same sub-pathways. Targeted follow-up studies are needed to confirm our observations and elucidate mechanisms related to how storage in different thermal conditions differentially affects metabolites in the same sub-pathways.

Our findings have implications for planning and interpreting untargeted metabolomics studies using feline serum. For metabolites with high stability, including those in the benzoate; glycine, serine and threonine; tryptophan; chemical (xenobiotics); acetylated peptide, and primary bile acid sub-pathways, short-term (< 6 months) storage at -20°C is expected to yield results comparable to samples stored at -80°C. Conversely, storage at -80°C would be preferred when the targets of an investigation include fatty acids, glutathione metabolites, and gamma-glutamyl amino acids. Accordingly, investigators should exercise caution in interpreting changes in these biochemicals in samples that were stored at -20°C.

There were several limitations to this study. First, we used the -80° samples stored for 12 months as a reference group for statistical comparisons. This assumes there are no significant changes in metabolite profiles when serum is stored at -80°C. Assessment of serum metabolite stability during storage at -80°C was not an objective of this investigation, however we did observe differences in the relative abundance of some serum metabolites (e.g. aspartate, glutamate) between samples stored at -80°C and those stored at -20°C. These differences could be explained by instability of the metabolites of interest, or as an effect of degradation of other precursor metabolites into the analytes of interest. As we did not analyze serum samples at baseline (time = 0 months), we cannot exclude the possibility that there was degradation of serum metabolites during storage at -80°C. In other studies, the stability of plasma metabolites stored at -80°C for up to 30 months has been established using untargeted nuclear magnetic resonance (NMR) [20]. As this previous study utilized different analytic methods (NMR vs HPLC-MS) and sample types (plasma vs serum) than our investigation, the extent to which these findings apply to the present study is unknown. It is plausible that changes in the relative abundances of serum metabolites occurred during storage of our samples for 1 year at -80°C. Nonetheless, storage at -80°C is expected to maintain the stability of serum samples for extended periods, freezers capable of maintaining temperatures of -80°C are available in most research laboratories, and storage at -80°C is a widely used method of samples preservation. For these reasons, we considered storage at -80°C for 1 year to be the reference “standard” for this investigation. Additional studies are required to determine the stability of feline serum metabolite profiles in samples stored for extended periods at -80°C. Second, this study was limited by the small number of cats. We cannot exclude the possibility that our findings would have been different using a larger cohort of cats. Third, we only assessed a limited number of pre-analytic storage conditions, as our objective was to understand whether serum stored in a standard clinical freezer would provide adequate sample preservation for untargeted metabolomics studies. Finally, serum used in this investigation was subjected to an incubation at room temperature to allow the whole blood to clot prior to centrifugation to separate the serum. It is likely that relative abundances of serum metabolites were affected by this incubation period. Using plasma would have allowed for the blood samples to have been processed and frozen more rapidly and reduced variation due to room temperature incubation. Serum is the most common biofluid collected in veterinary clinics and plasma is rarely used, thus we elected to use serum for this study. Future investigations should include larger numbers of cats, a more diverse set of pre-analytic conditions, and compare plasma to serum profiles.

## Conclusions

Understanding the pre-analytic stability of metabolomes during storage is critical to avoid errors in interpretation of results from untargeted metabolomics studies conducted in clinical settings. The purpose of this study was to assess the stability of serum metabolomes in samples exposed pre-analytic storage at -20°C and compare them to samples stored at -80°C. We conclude that the individual cat of origin, rather than storage condition, was the most important component of variation in metabolite profiles among serum samples. Most serum metabolites in cats were classified with moderate-excellent stability based on intraclass correlation analysis. Among serum metabolites with relative abundances that varied significantly with storage at -20°C, and those classified with poor stability, we identified many metabolites that are involved in redox reactions (glutathione metabolites, gamma-glutamyl amino acids) or are substrates for oxidation (fatty acids), these findings suggest that at storage of serum at -20°C is associated with increased sample oxidation. Investigators should exercise caution when interpreting untargeted serum metabolome profiles in cats in samples stored at -20°C, especially with respect to glutathione metabolites, gamma-glutamyl amino acids, and fatty acids. By providing evidence of the stability of serum metabolites during pre-analytic storage and a catalog of metabolites that vary during storage at -20°C, this investigation provides valuable insights that could aid other investigators in planning and interpreting studies of serum metabolomes in cats.

## Supporting information

S1 File (ZIP)
