# Supplementary material for: Untargeted metabolomics reveals the effects of pre-analytic storage on serum metabolite profiles from healthy cats
Source: PLoS One. 2024 May 30;19(5):e0303500. doi: 10.1371/journal.pone.0303500 (PMC11139287; doi:10.1371/journal.pone.0303500)
Supplement: S1 File — (ZIP) [file pone.0303500.s001.zip › Supporting Info/S5File.pdf]

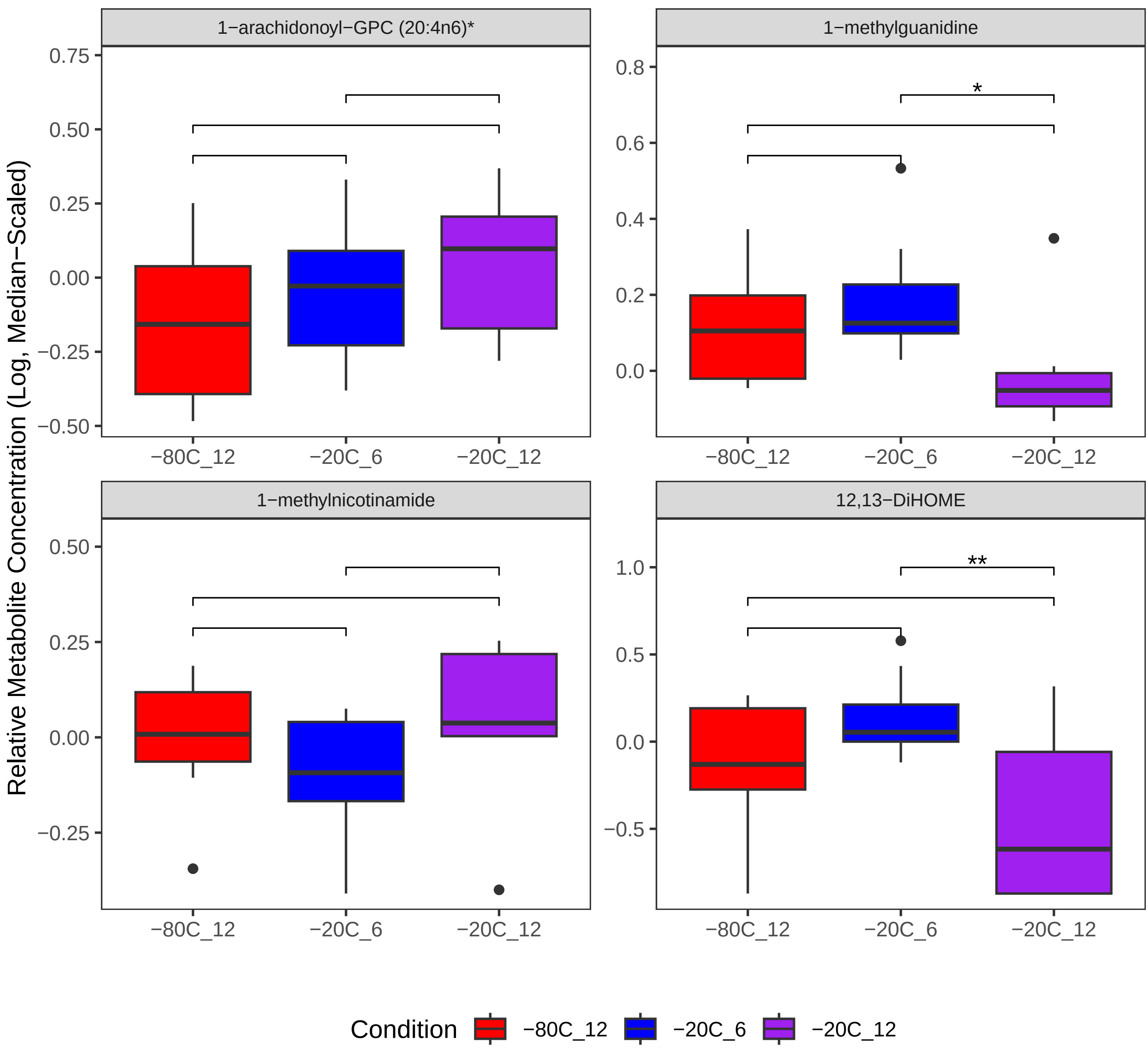
1−arachidonoyl−GPC (20:4n6)*
1−methylguanidine
1−methylnicotinamide
12,13−DiHOME
Relative Metabolite Concentration (Log, Median−Scaled)
0.75
0.50
0.25
0.00
−0.25
−0.50
0.8
0.6
0.4
0.2
0.0
1.0
0.5
−0.5
*
**
−80C_12
−20C_6
−20C_12
Condition
−80C_12
−20C_6
−20C_12

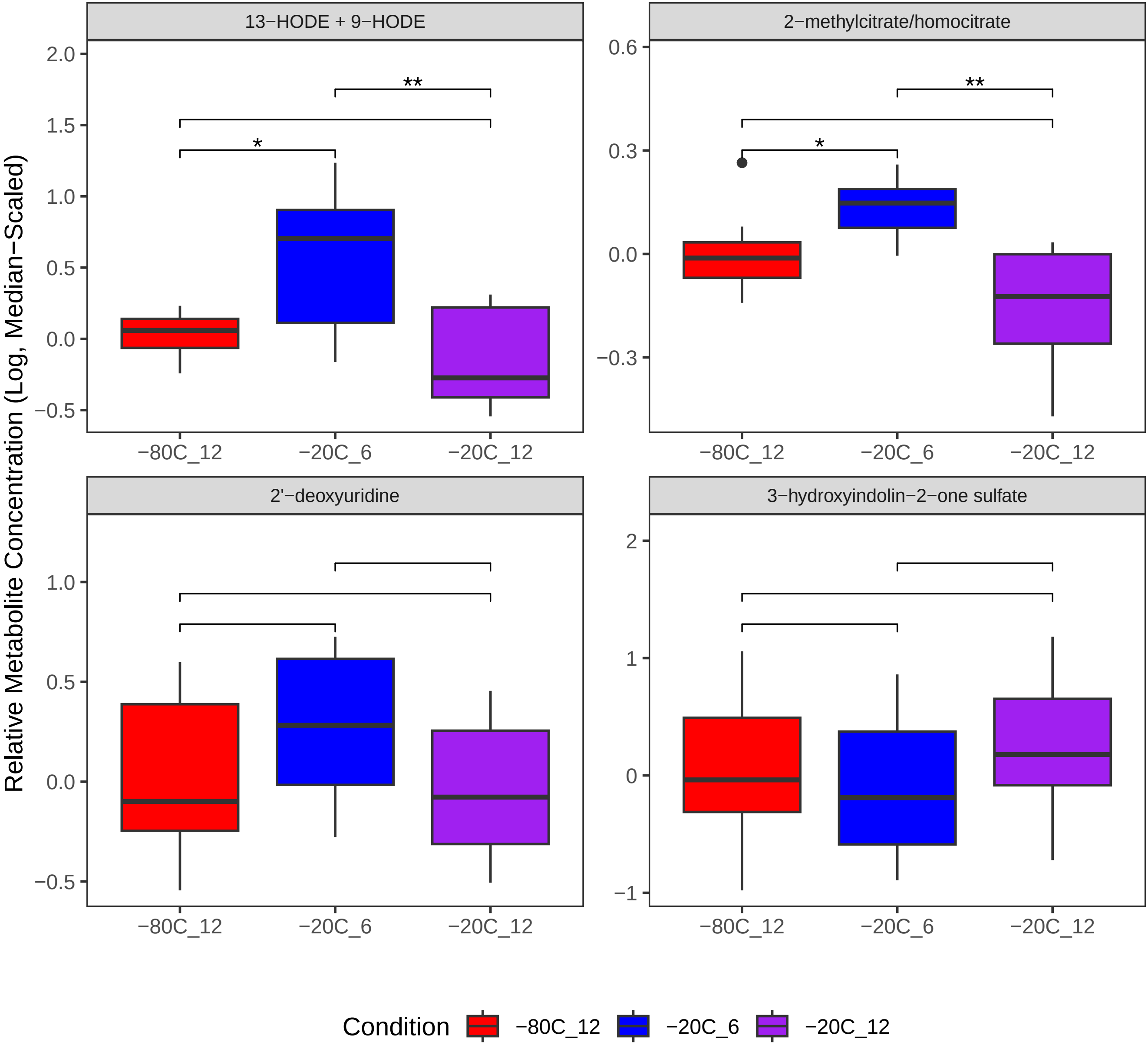
13−HODE + 9−HODE
2−methylcitrate/homocitrate
2'−deoxyuridine
3−hydroxyindolin−2−one sulfate
Relative Metabolite Concentration (Log, Median−Scaled)
−80C_12
−20C_6
−20C_12
*
**
Condition
−80C_12
−20C_6
−20C_12

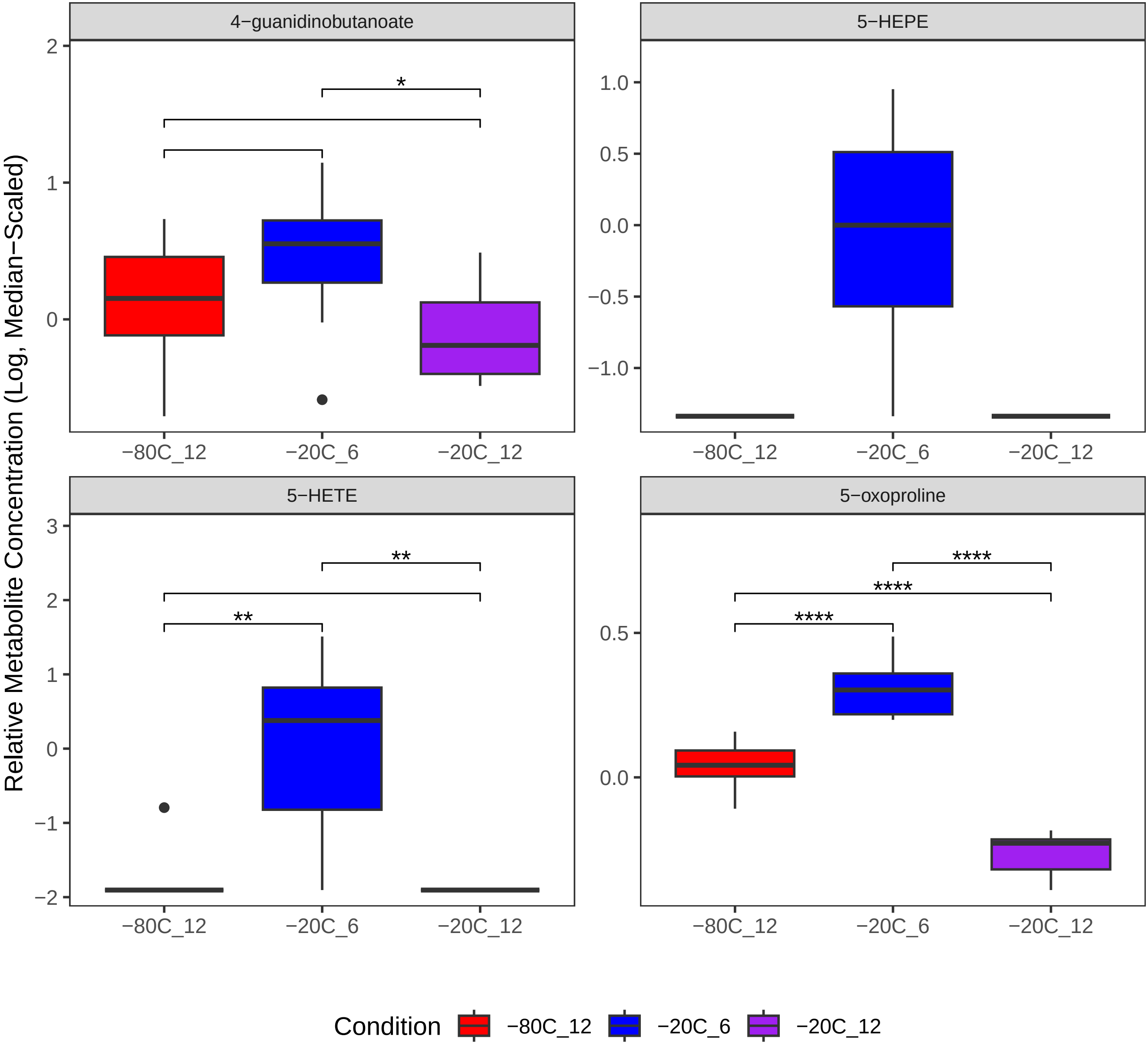
4−guanidinobutanoate
5−HEPE
5−HETE
5−oxoproline
Relative Metabolite Concentration (Log, Median−Scaled)
*
**
**
****
****
****
−80C_12
−20C_6
−20C_12
Condition
−80C_12
−20C_6
−20C_12

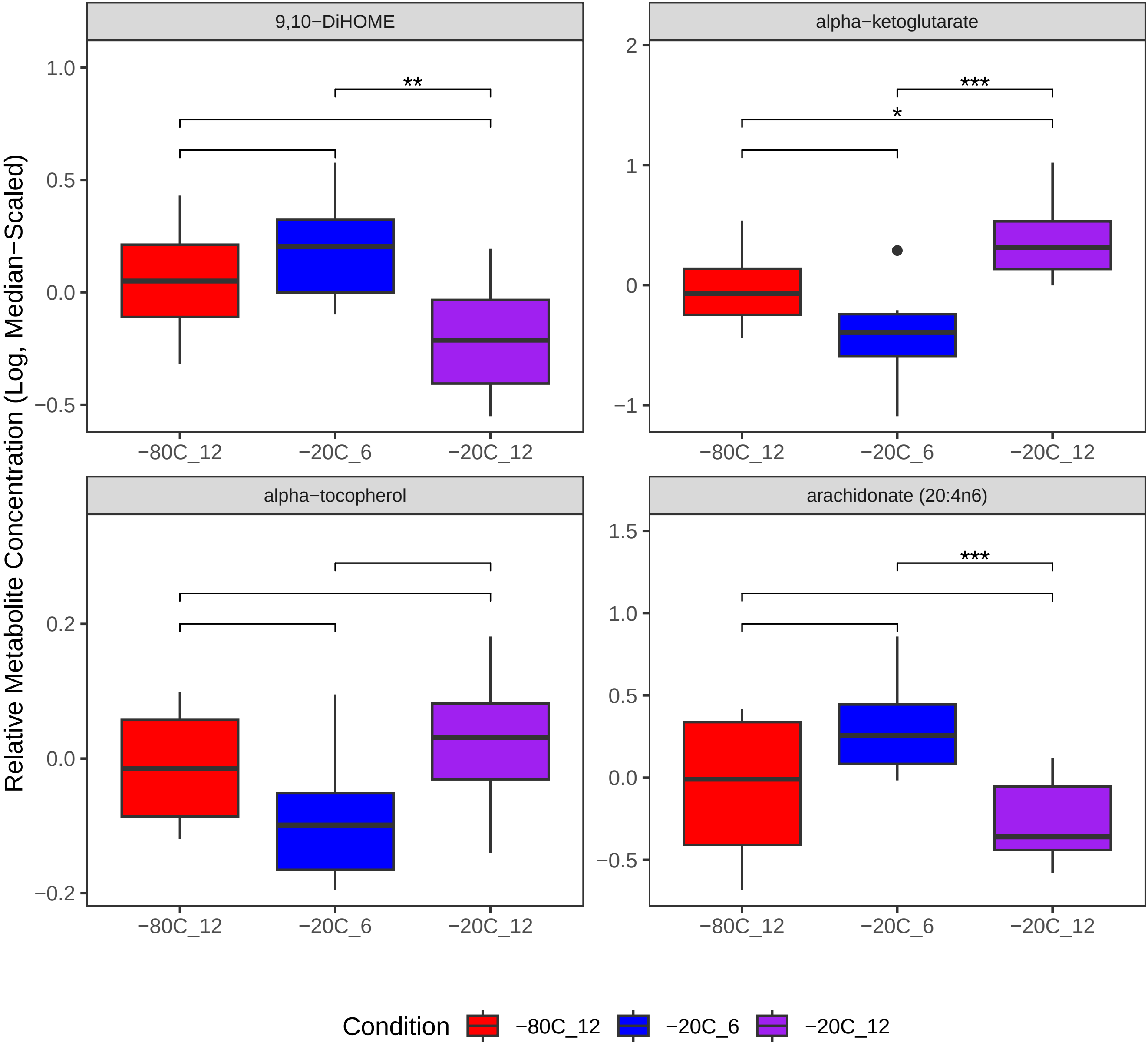
9,10−DiHOME
alpha−ketoglutarate
alpha−tocopherol
arachidonate (20:4n6)
Relative Metabolite Concentration (Log, Median−Scaled)
−80C_12
−20C_6
−20C_12
**
***
*
***
Condition
−80C_12
−20C_6
−20C_12

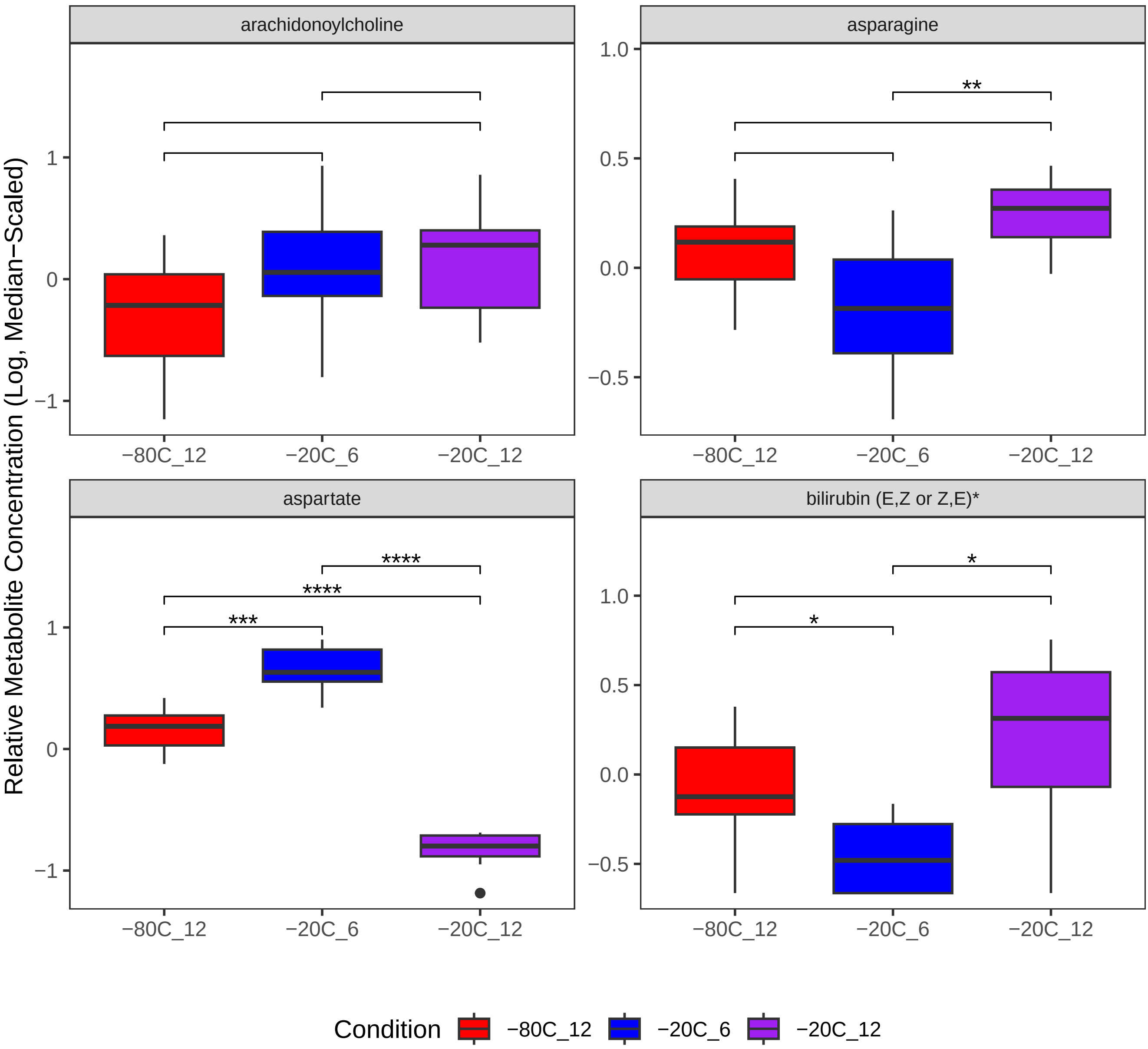
arachidonoylcholine
asparagine
aspartate
bilirubin (E,Z or Z,E)*
Relative Metabolite Concentration (Log, Median−Scaled)
−80C_12
−20C_6
−20C_12
**
****
****
***
*
*
*
Condition
−80C_12
−20C_6
−20C_12

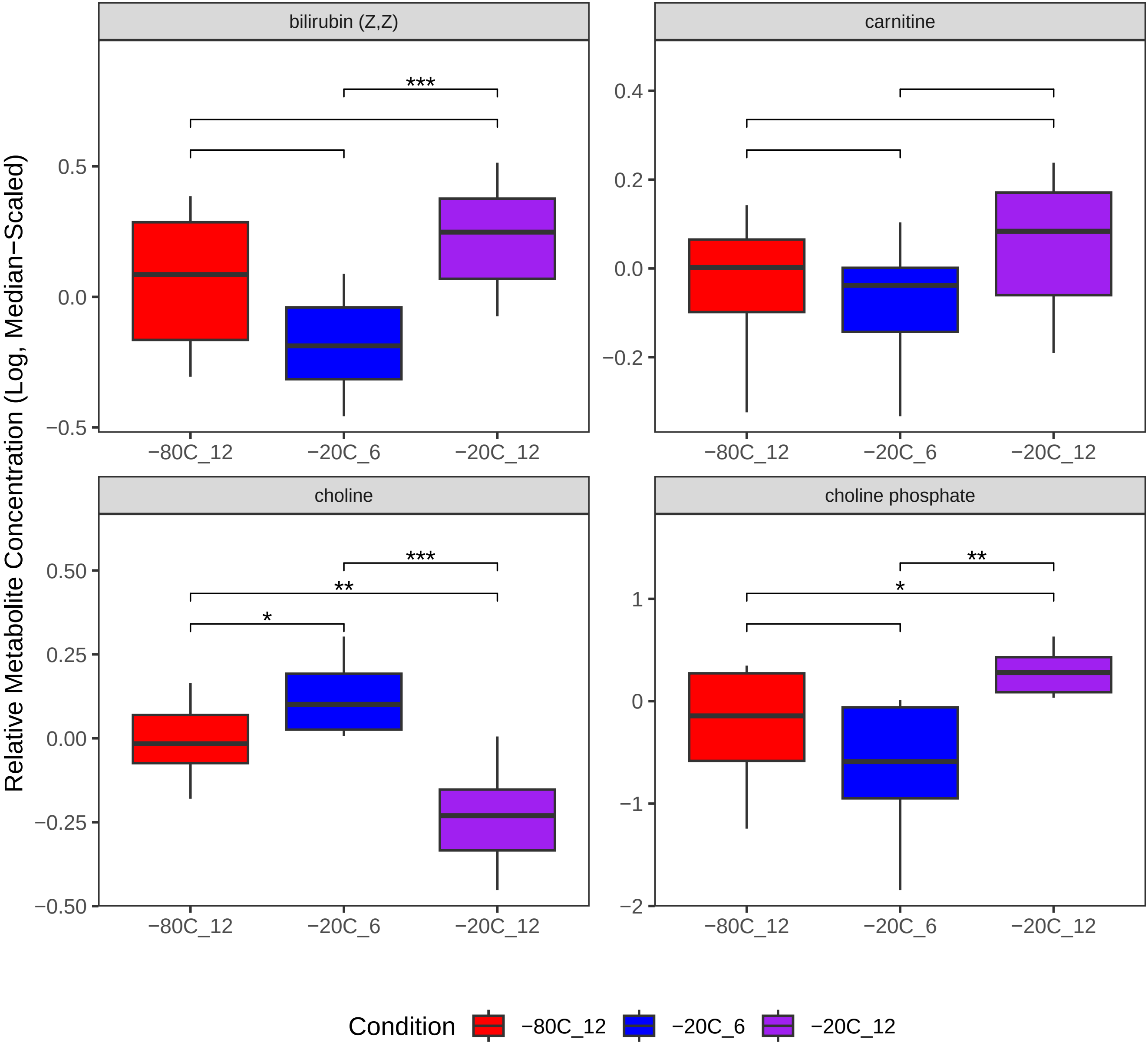
bilirubin (Z,Z)
carnitine
choline
choline phosphate
Relative Metabolite Concentration (Log, Median−Scaled)
−80C_12
−20C_6
−20C_12
***
**
*
Condition

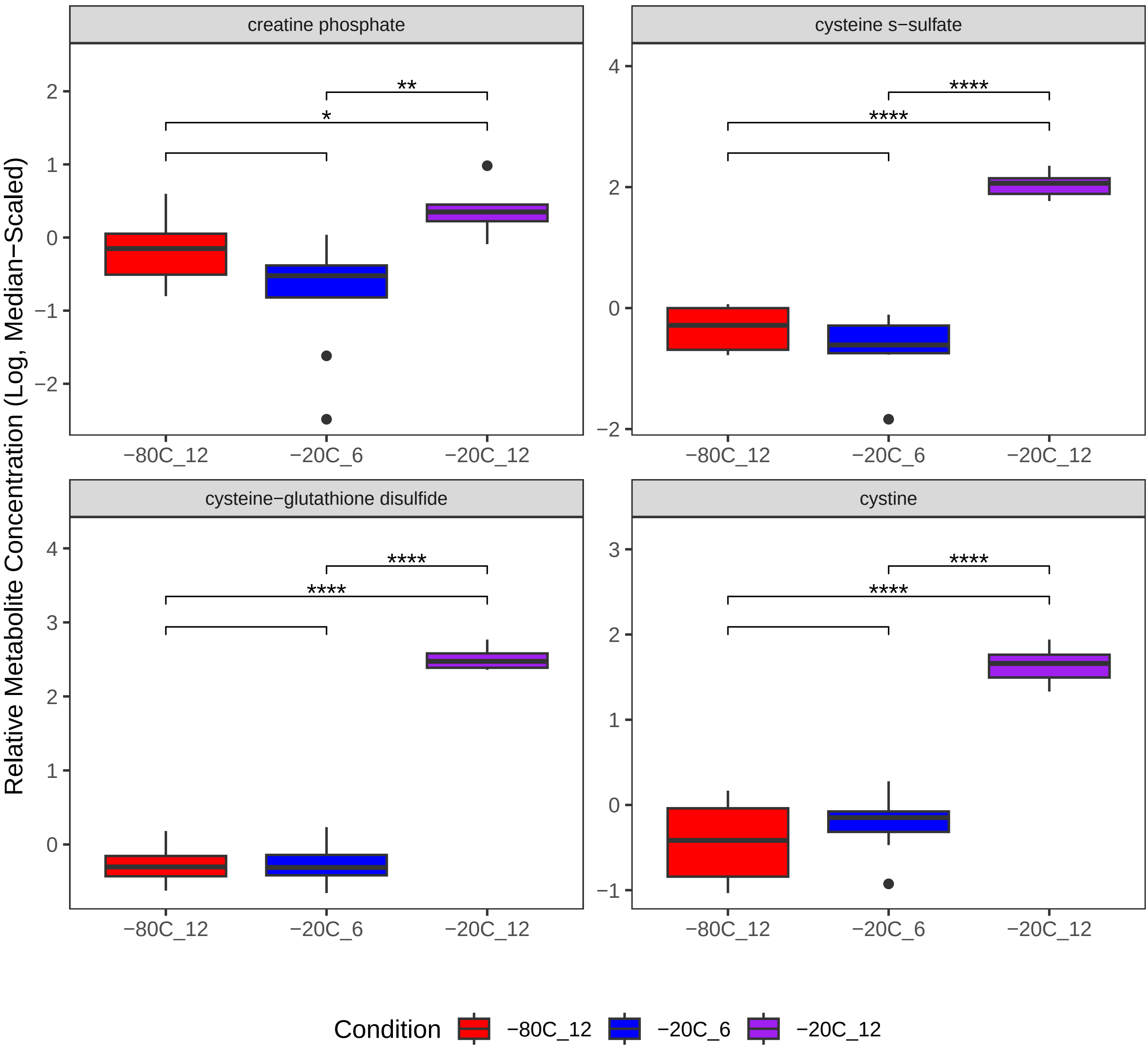
creatine phosphate
cysteine s−sulfate
cysteine−glutathione disulfide
cystine
Relative Metabolite Concentration (Log, Median−Scaled)
−80C_12
−20C_6
−20C_12
*
**
****
Condition

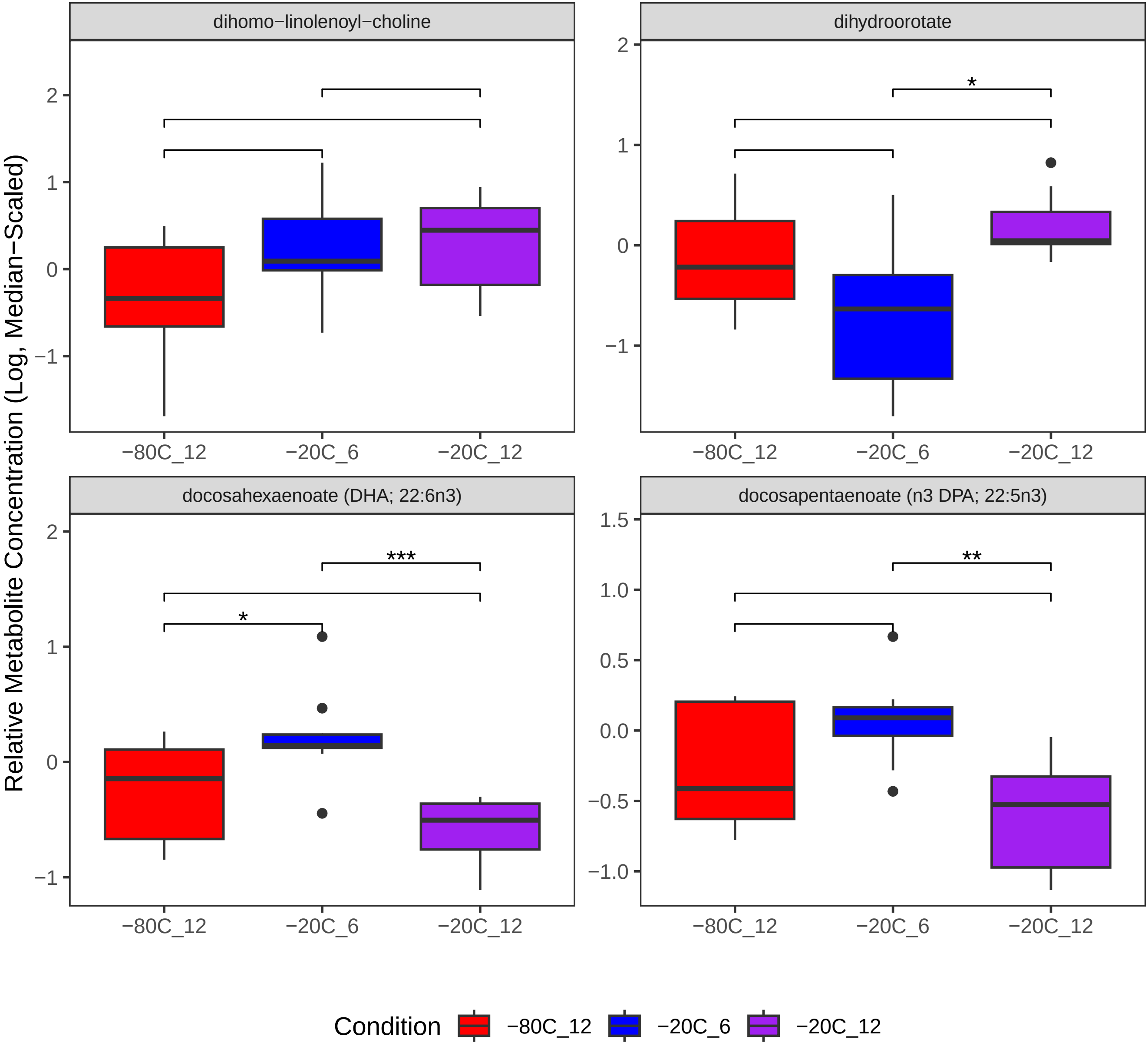

dihomo−linolenoyl−choline
dihydroorotate
docosahexaenoate (DHA; 22:6n3)
docosapentaenoate (n3 DPA; 22:5n3)
Relative Metabolite Concentration (Log, Median−Scaled)
−80C_12
−20C_6
−20C_12
*
***
*
**
Condition
−80C_12
−20C_6
−20C_12

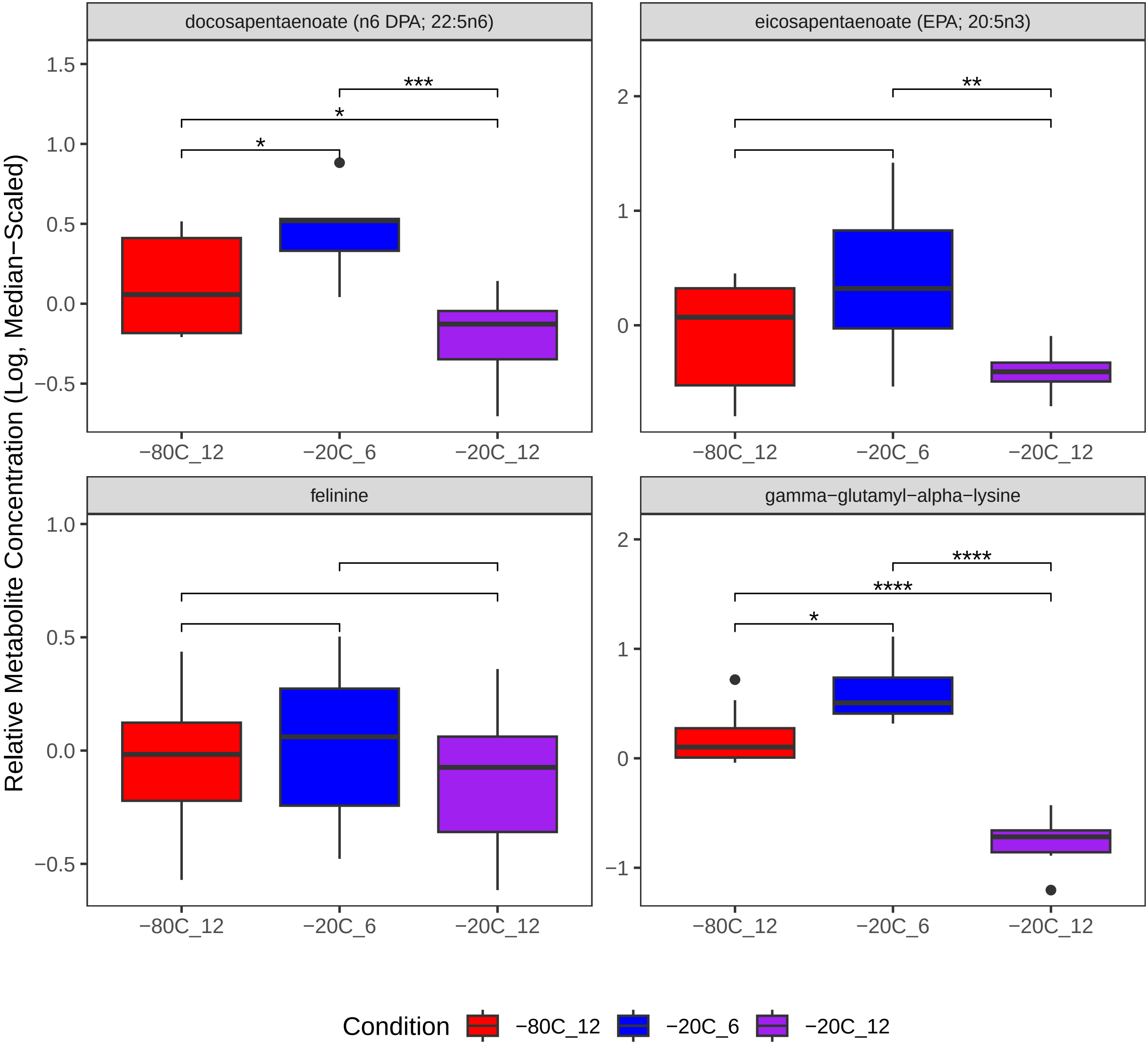

docosapentaenoate (n6 DPA; 22:5n6)
eicosapentaenoate (EPA; 20:5n3)
felinine
gamma−glutamyl−alpha−lysine
Relative Metabolite Concentration (Log, Median−Scaled)
−80C_12
−20C_6
−20C_12
***
*
*
**
****
****
*
1.5
1.0
0.5
0.0
−0.5
2
1
0
−1
Condition
−80C_12
−20C_6
−20C_12

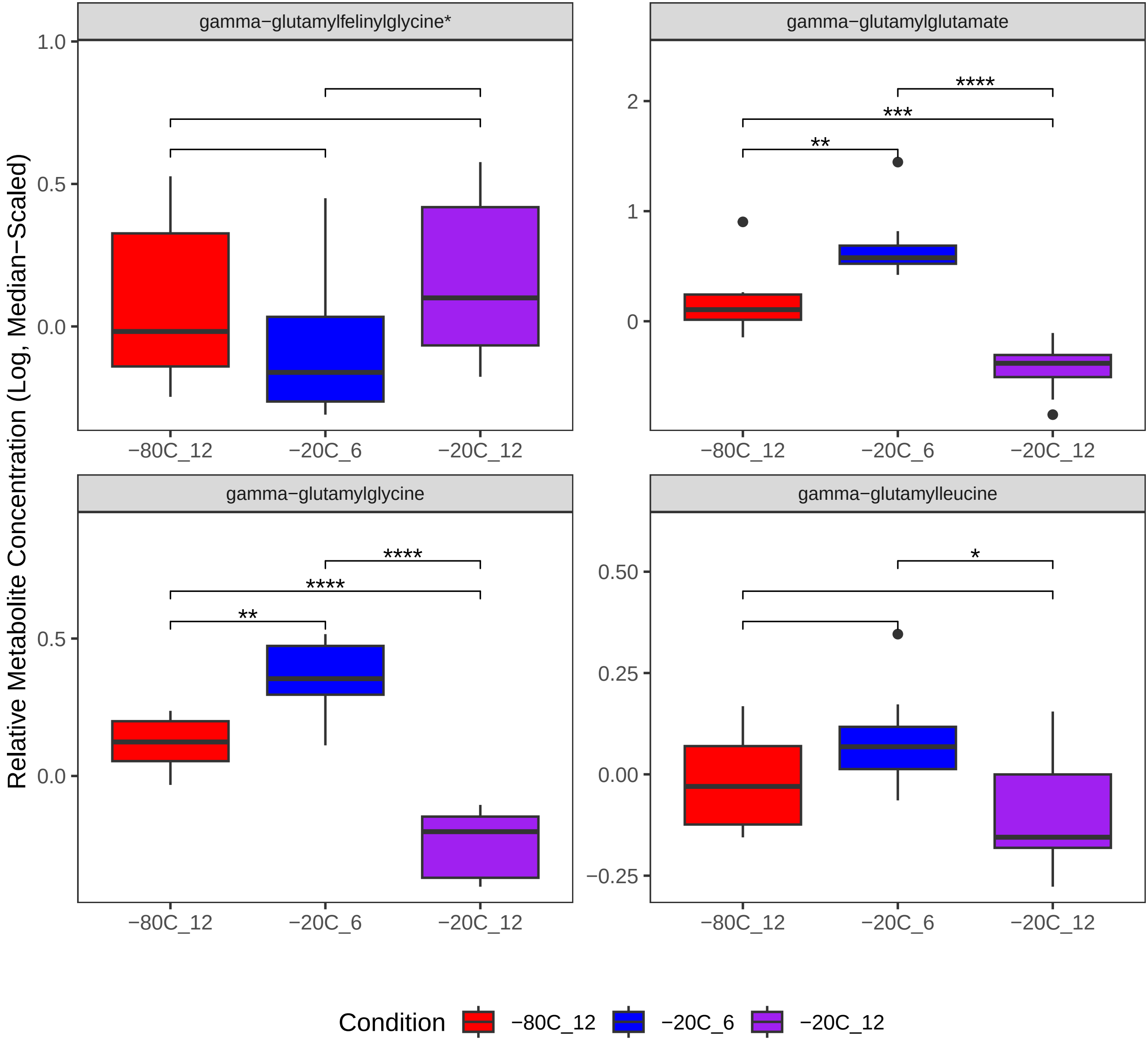
gamma−glutamylfelinylglycine*
gamma−glutamylglutamate
gamma−glutamylglycine
gamma−glutamylleucine
Relative Metabolite Concentration (Log, Median−Scaled)
1.0
0.5
0.0
2
1
0
0.50
0.25
0.00
−0.25
****
***
**
*
−80C_12
−20C_6
−20C_12
Condition
−80C_12
−20C_6
−20C_12

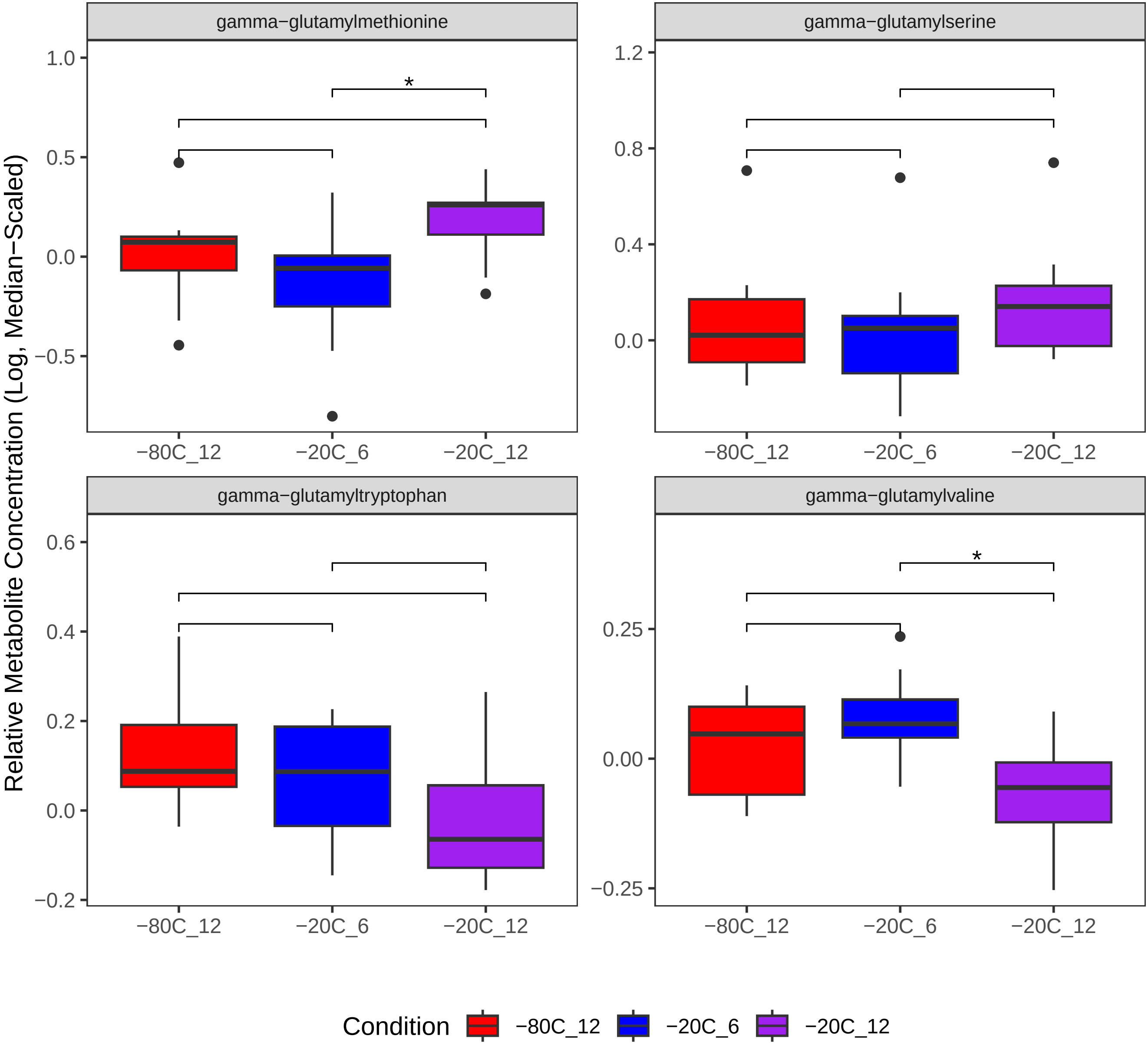
gamma−glutamylmethionine
gamma−glutamylserine
gamma−glutamyltryptophan
gamma−glutamylvaline
Relative Metabolite Concentration (Log, Median−Scaled)
1.0
0.5
0.0
−0.5
1.2
0.8
0.4
0.0
0.6
0.4
0.2
0.0
−0.2
0.25
0.00
−0.25
*
*
−80C_12
−20C_6
−20C_12
Condition
−80C_12
−20C_6
−20C_12

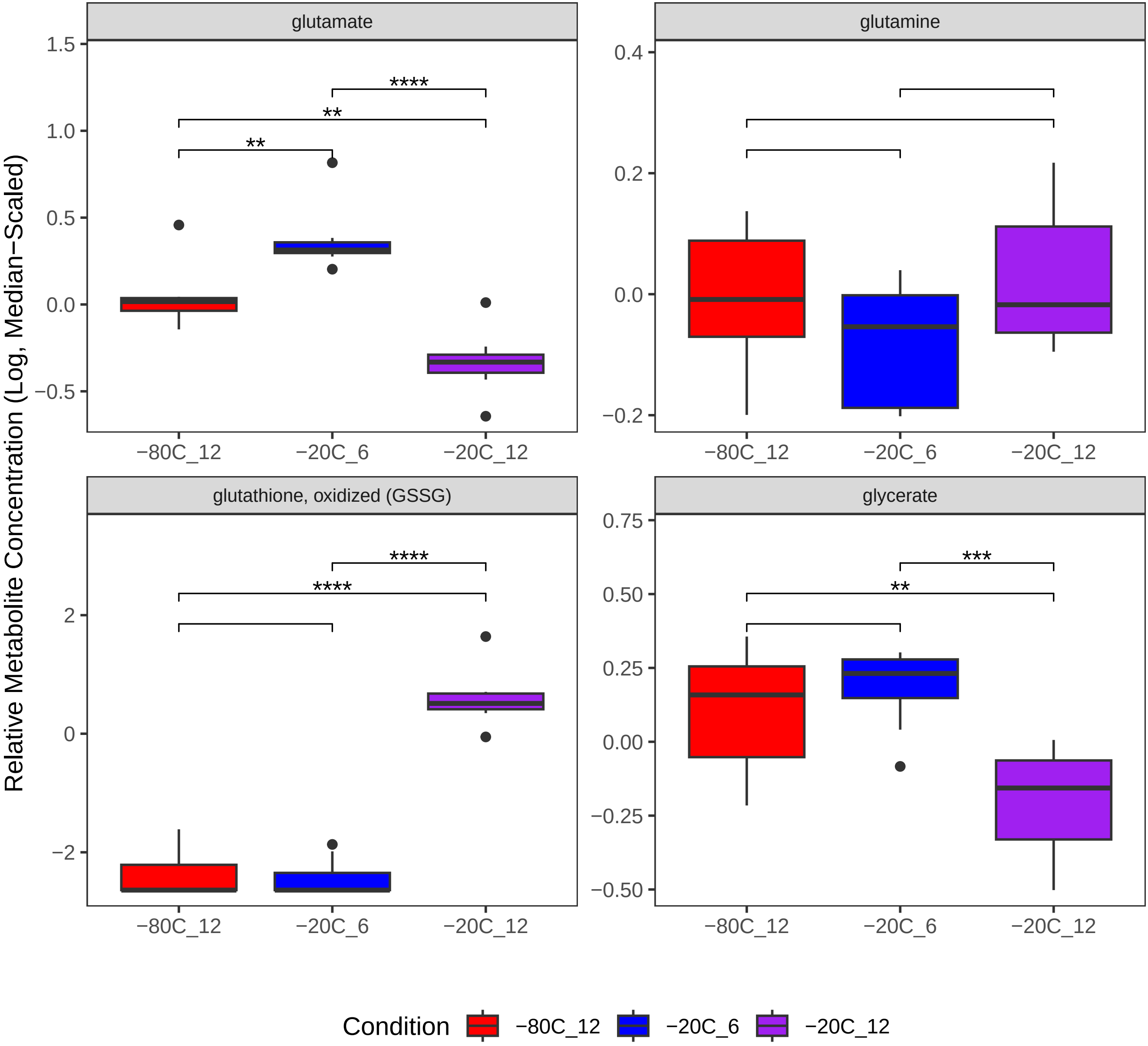
Relative Metabolite Concentration (Log, Median−Scaled)
glutamate
1.5
1.0
0.5
0.0
−0.5
****
**
**
glutamine
0.4
0.2
0.0
−0.2
glutathione, oxidized (GSSG)
2
0
−2
****
****
glycerate
0.75
0.50
0.25
0.00
−0.25
−0.50
***
**
−80C_12
−20C_6
−20C_12
Condition
−80C_12
−20C_6
−20C_12

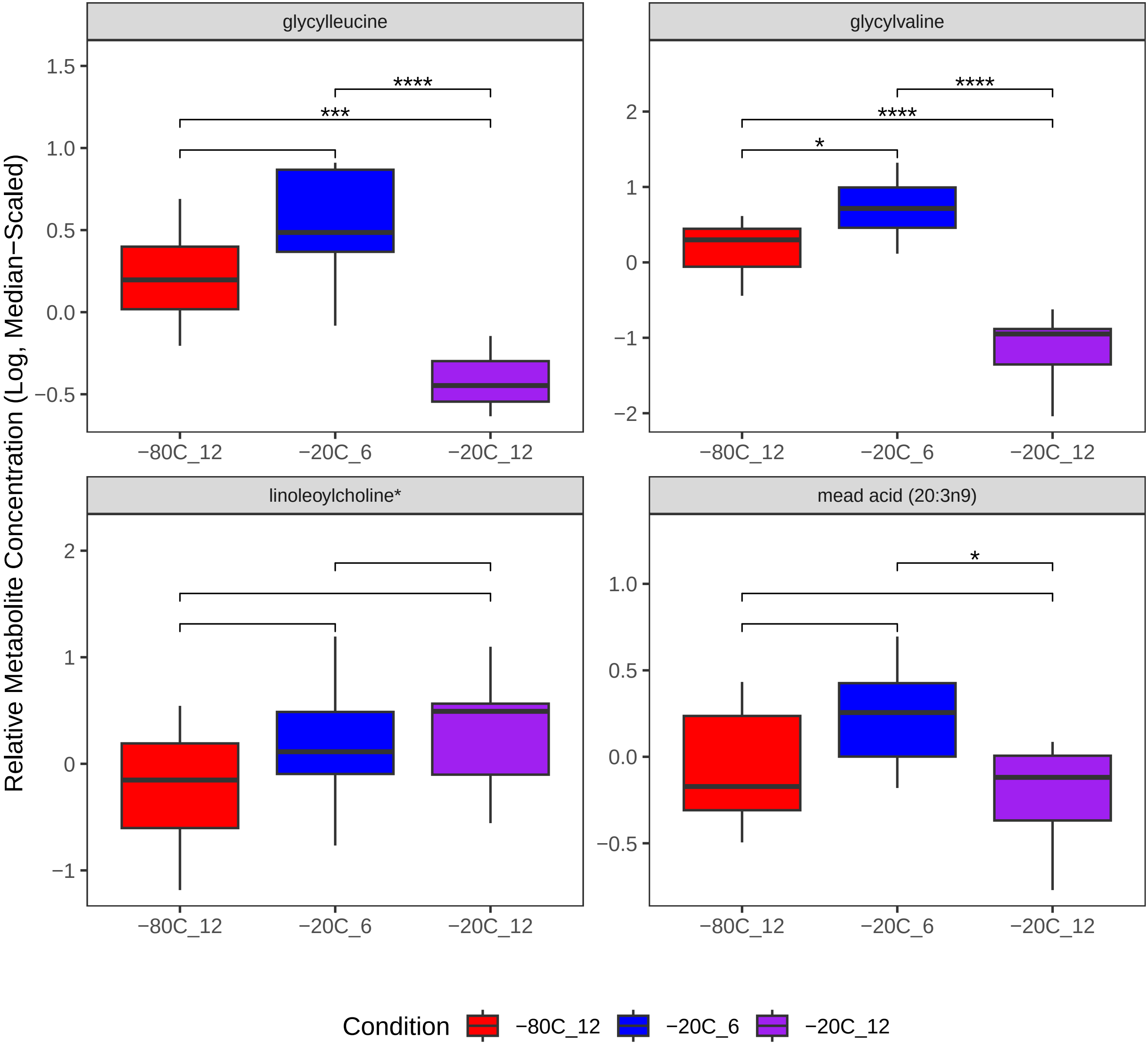
glycylleucine
glycylvaline
linoleoylcholine*
mead acid (20:3n9)
****
***
*
****
****
*
1.5
1.0
0.5
0.0
−0.5
2
1
0
−1
−2
1.0
0.5
0.0
−0.5
−80C_12
−20C_6
−20C_12
Relative Metabolite Concentration (Log, Median−Scaled)
Condition
−80C_12
−20C_6
−20C_12

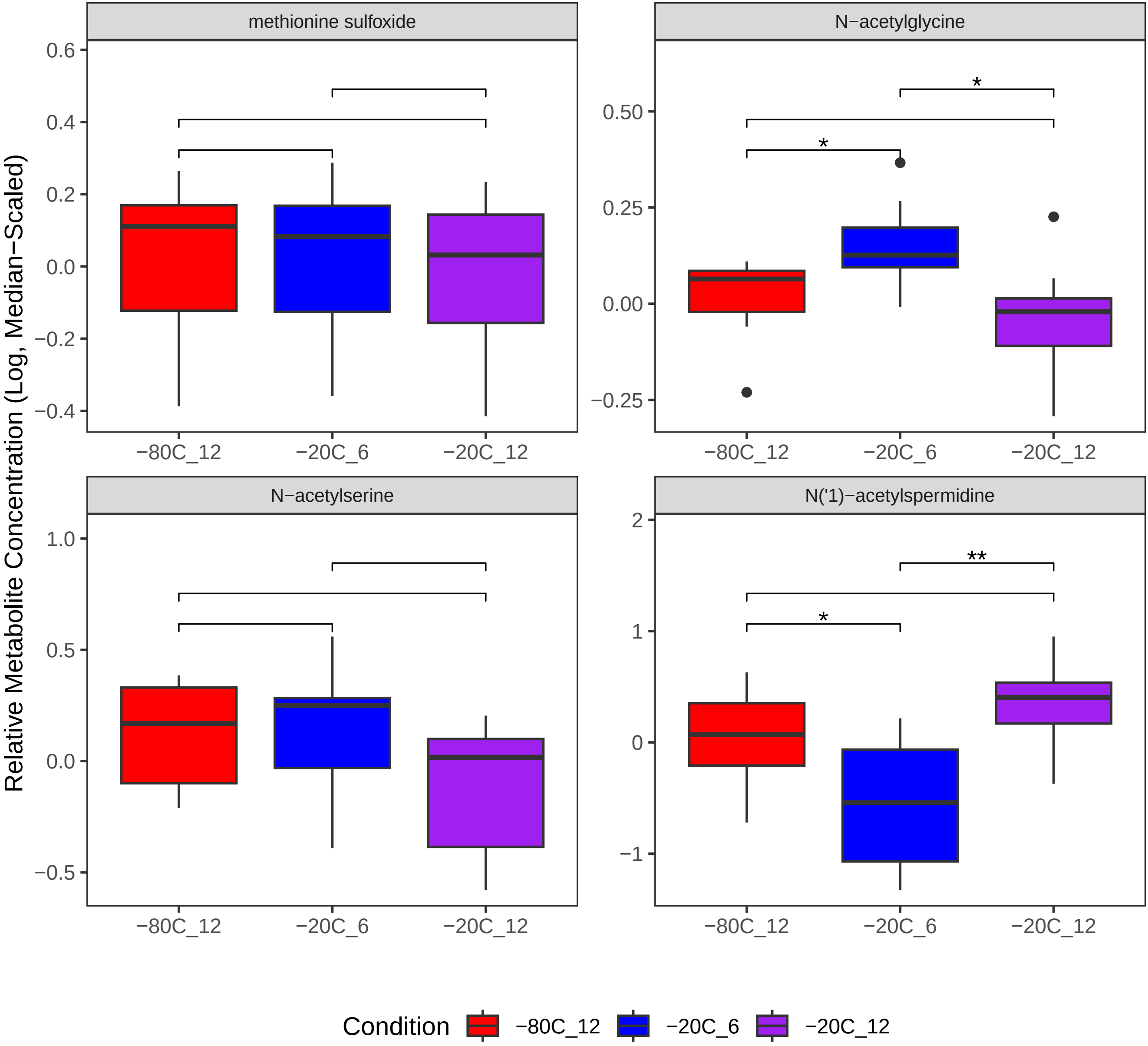
methionine sulfoxide
N−acetylglycine
N−acetylserine
N('1)−acetylspermidine
Relative Metabolite Concentration (Log, Median−Scaled)
−80C_12
−20C_6
−20C_12
*
**
Condition
−80C_12
−20C_6
−20C_12

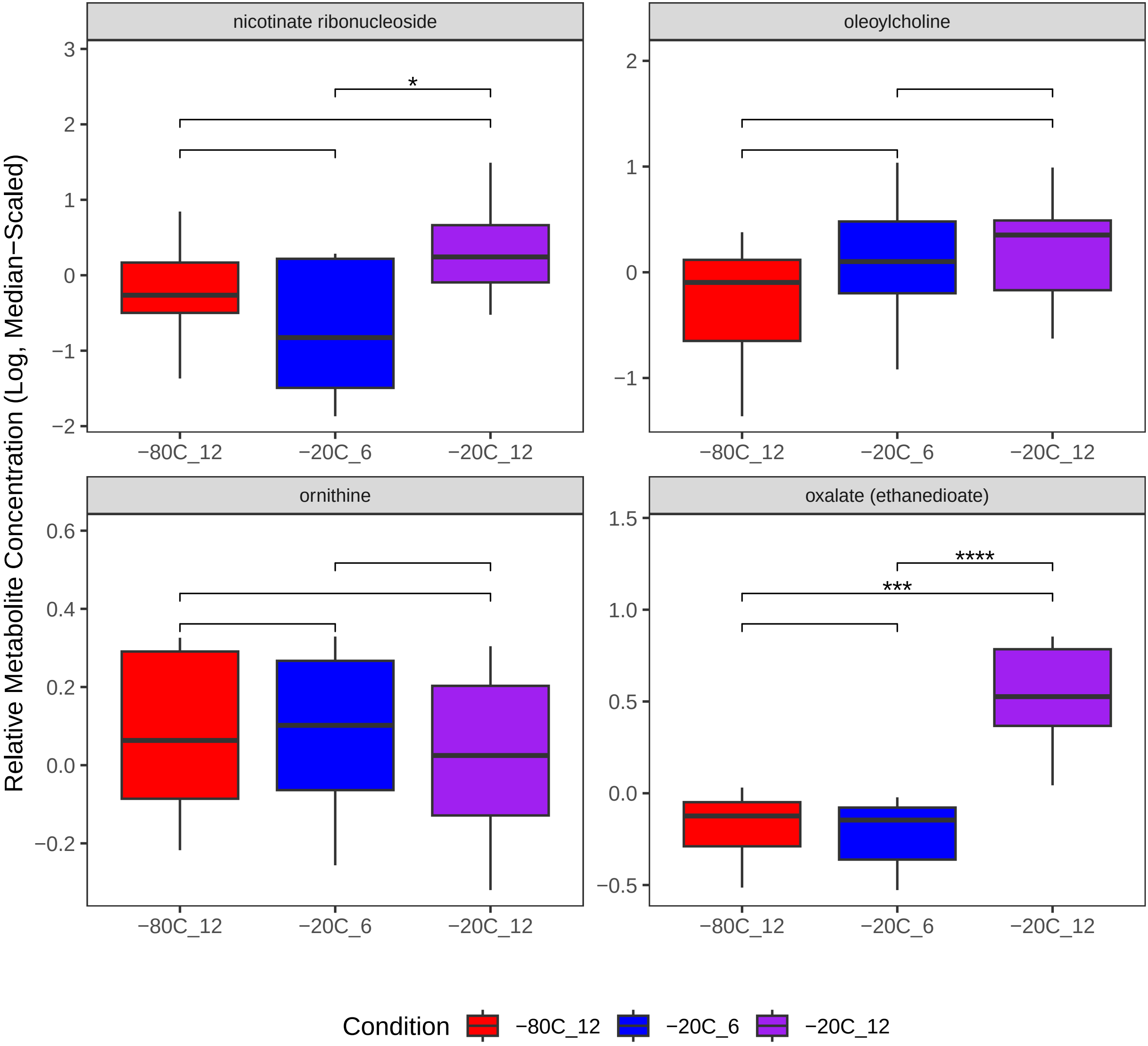
nicotinate ribonucleoside
oleoylcholine
ornithine
oxalate (ethanedioate)
Relative Metabolite Concentration (Log, Median−Scaled)
*
***
****
3
2
1
0
−1
−2
1.5
1.0
0.5
0.0
−0.5
0.6
0.4
0.2
−0.2
−80C_12
−20C_6
−20C_12
Condition

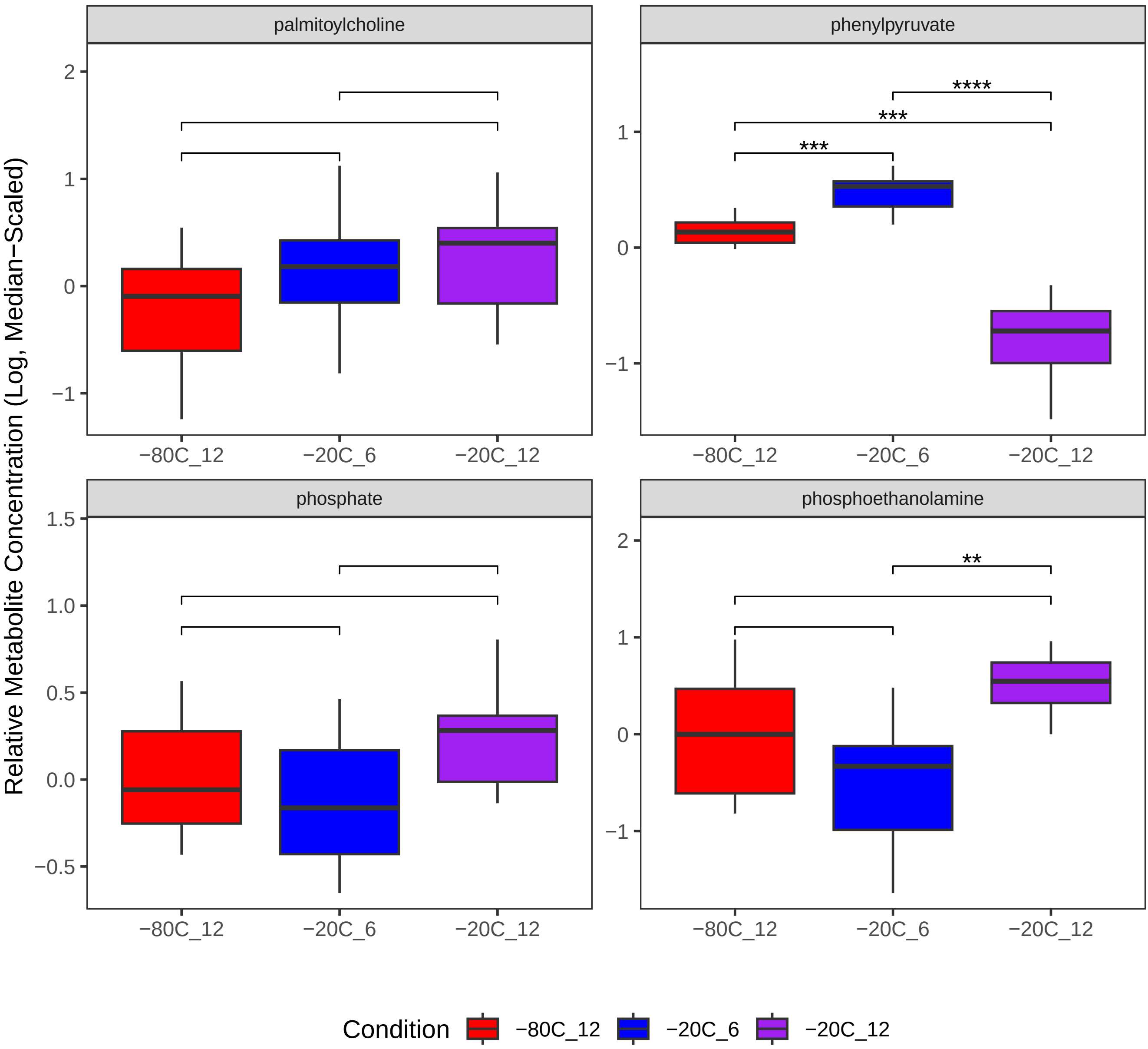
palmitoylcholine
phenylpyruvate
phosphate
phosphoethanolamine
Relative Metabolite Concentration (Log, Median−Scaled)
−80C_12
−20C_6
−20C_12
****
***
***
**
Condition
−80C_12
−20C_6
−20C_12

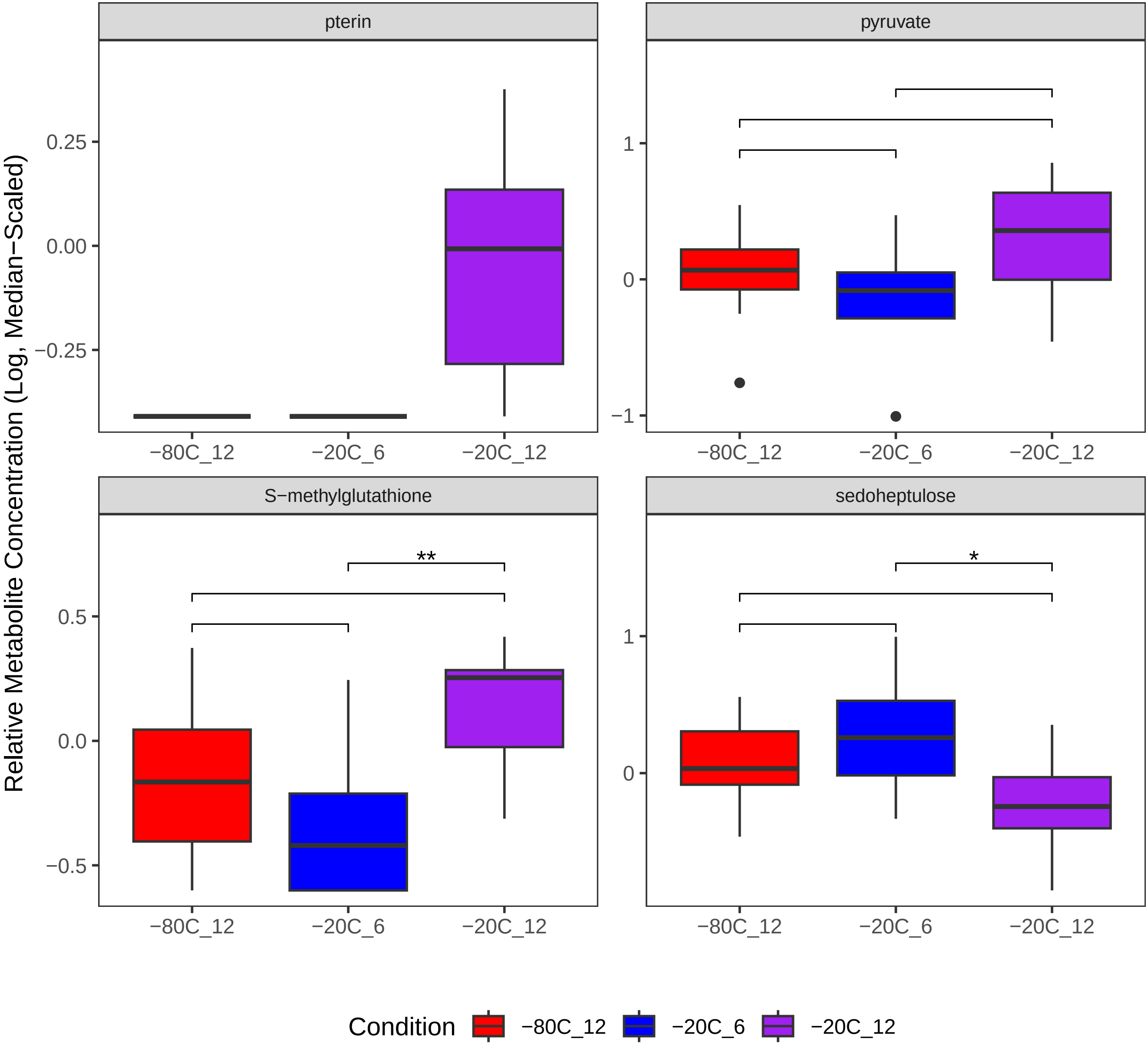
pterin
pyruvate
S−methylglutathione
sedoheptulose
Relative Metabolite Concentration (Log, Median−Scaled)
0.25
0.00
−0.25
1
0
−1
0.5
0.0
−0.5
**
*
−80C_12
−20C_6
−20C_12
Condition
−80C_12
−20C_6
−20C_12

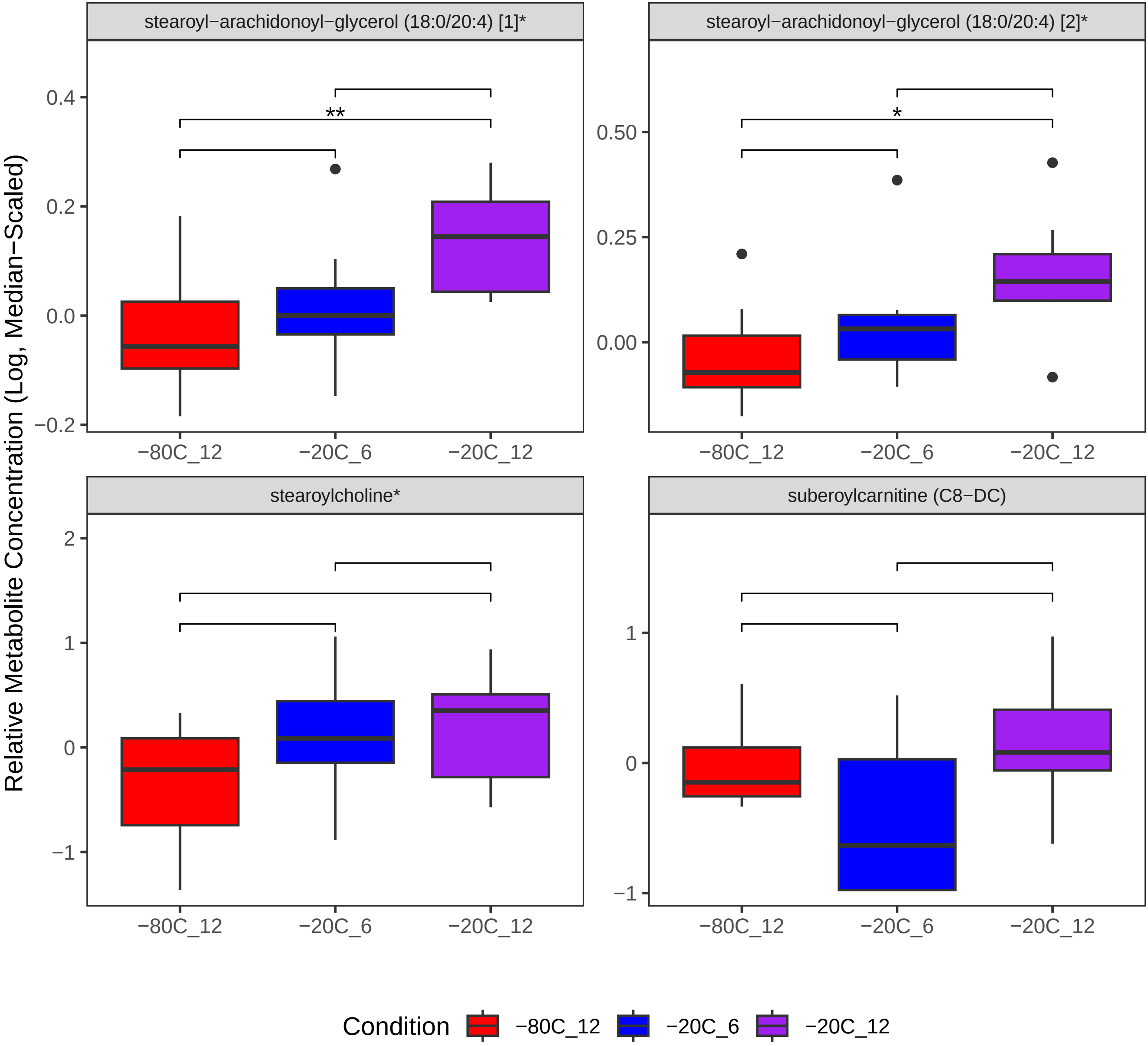
stearoyl−arachidonoyl−glycerol (18:0/20:4) [1]*
stearoyl−arachidonoyl−glycerol (18:0/20:4) [2]*
stearoylcholine*
suberoylcarnitine (C8−DC)
**
*
Relative Metabolite Concentration (Log, Median−Scaled)
0.4
0.2
0.0
−0.2
0.50
0.25
0.00
2
1
0
−1
−80C_12
−20C_6
−20C_12
Condition

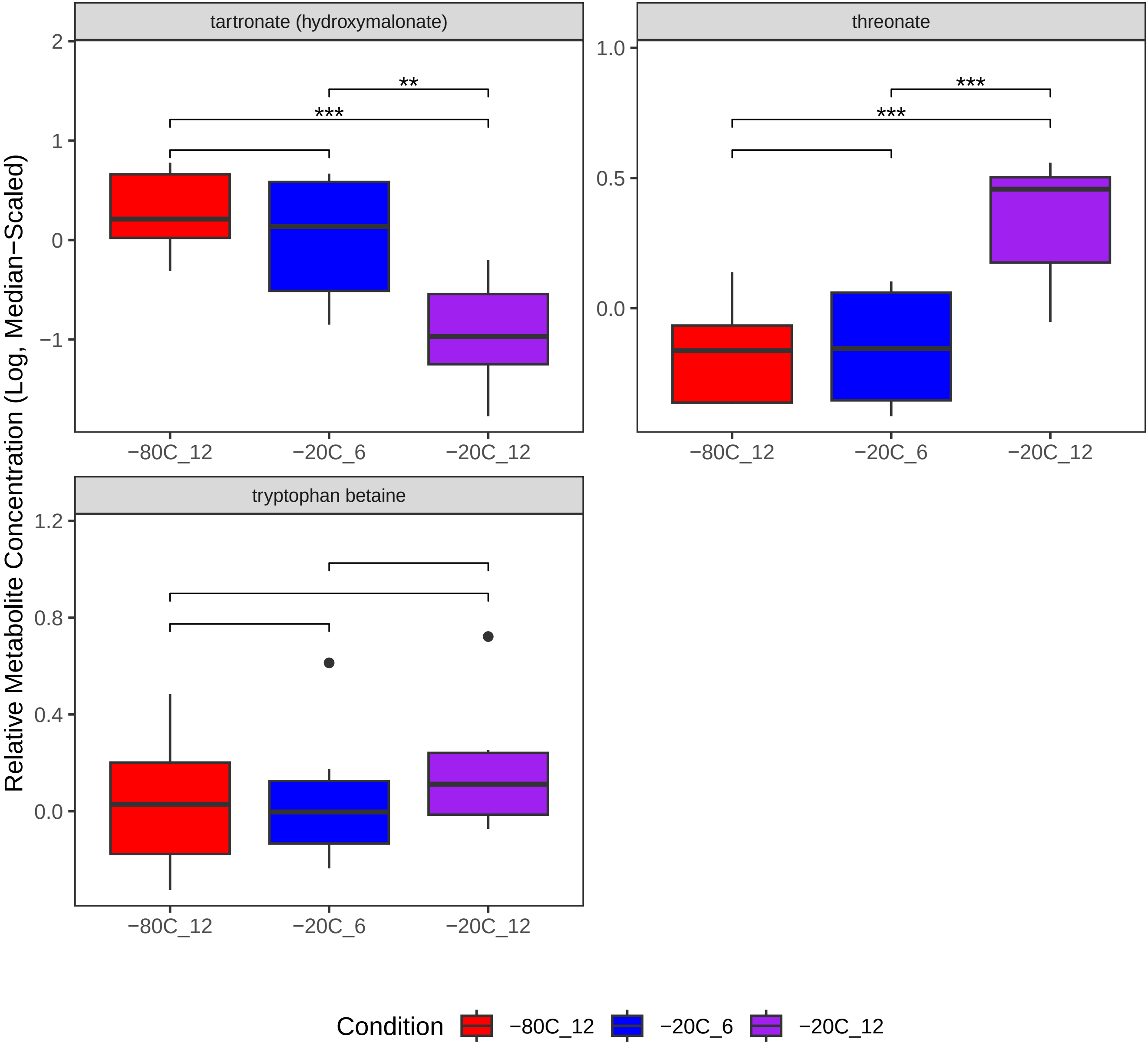
tartronate (hydroxymalonate)
threonate
tryptophan betaine
**
***
***
***
−80C_12
−20C_6
−20C_12
Relative Metabolite Concentration (Log, Median−Scaled)
Condition
−80C_12
−20C_6
−20C_12
