# Supplementary material for: Untargeted metabolomics reveals the effects of pre-analytic storage on serum metabolite profiles from healthy cats
Source: PLoS One. 2024 May 30;19(5):e0303500. doi: 10.1371/journal.pone.0303500 (PMC11139287; doi:10.1371/journal.pone.0303500)
Supplement: S1 File — (ZIP) [file pone.0303500.s001.zip › Supporting Info/S1File.docx]

**Supplemental Information: Chromatographic Conditions**

**LC/MS Positive (LC/MS/MS Pos Early), Optimized for Hydrophilic Compounds**

Column: Waters BEH C18 1.7um 2.1 x 100mm
Mobile Phase A: 0.1% formic acid and 0.05% PFPA in water, pH ~2.5

Mobile Phase B: 0.1% formic acid and 0.05% PFPA in methanol, pH ~2.5

Flow Rate: 0.35 mL/min

Gradient Elution: Linear gradient from 5% B to 80% B over 3.35 minutes.

Instrument Performance Standards: d7-glucose, d5-glutamine, d2-threonine, d5-hippuric acid d3-methionine, d3-leucine, Br-phenylalanine

Process Assessment Standards: fluorophenylglycine, chlorophenylalanine

**LC/MS Positive (LC/MS/MS Pos Late), Optimized for Hydrophobic Compounds**

Column: Waters BEH C18 1.7um 2.1 x 100mm
Mobile Phase A: 0.1% formic acid and 0.05% PFPA in water, pH ~2.5

Mobile Phase B: 0.1% formic acid and 0.05% PFPA in 50% methanol/ 50% acetonitrile, pH ~2.5

Flow Rate: 0.60 mL/min

Gradient Elution: Linear gradient from 40% B to 99.5% B over 1.0 minute, hold 99.5% B for 2.4 minutes.

Instrument Performance Standards: Br-phenylalanine, d5-androstene, d9-progesterone d4-dioctyphthalate

Process Assessment Standards: d6-cholesterol chlorophenylalanine

**Chromatographic Conditions LC/MS Negative (LC/MS/MS Neg)**

Column: Waters BEH C18 1.7um 2.1 x 100mm
Mobile Phase A: 6.5 mM ammonium bicarbonate in water, pH 8

Mobile Phase B: 6.5 mM ammonium bicarbonate in 95% methanol/ 5% water

Flow Rate: 0.35 mL/min

Gradient Elution: Linear gradient from 0.5 to 70% B over 4.0 minutes, then rapid gradient to 99% B in 0.5 minutes.

Instrument Performance Standards: d7-glucose, d3-methionine, d3-leucine, d8-phenyulalanine, d5-tryptophan, Br-phenylalanine, d15-octanoic acid, d19-decanoic acid, d27-tetradecanoic acid, d35-octadecanoic, acid d2-eicosanoic acid

Process Assessment Standards: tridecanoic acid, chlorophenylalanine

**Chromatographic Conditions LC/MS Negative (LC/MS/MS Polar)**

Column: Waters BEH Amide 1.7um 2.1 x 150mm

Mobile Phase A: 10 mM ammonium formate in 15% water/5% methanol/80% acetonitrile (effective pH 10.16 with NH4OH)

Mobile Phase B: 10 mM ammonium formate in 50% water/ 50% acetonitrile (effective pH 10.60 with NH4OH)

Flow Rate: 0.50 mL/min

Gradient Elution: gradient from 50% B to 95% B in 2 minutes minutes.

Instrument Performance Standards: d35-octadecanoic acid, d5-indole acetate, Br-phenylalanine, d5-tryptophan, d4-tyrosine, d3-serine, d3-aspartic acid, d7-ornithine, d4-lysine

Process Assessment Standards: fluorophenylglycine, chlorophenylalanine
